# Bulky Lesion Bypass Requires Dpo4 Binding in Distinct Conformations

**DOI:** 10.1038/s41598-017-17643-0

**Published:** 2017-12-12

**Authors:** Pramodha S. Liyanage, Alice R. Walker, Alfonso Brenlla, G. Andrés Cisneros, Louis J. Romano, David Rueda

**Affiliations:** 1 Department of Chemistry, Wayne State University, Detroit, MI 48202 USA; 2 Department of Chemistry, University of North Texas, Denton, TX 76201 USA; 3 Molecular Virology, Department of Medicine, Imperial College London, Du Cane Road, London, W12 0NN UK; 4 Single Molecule Imaging Group, MRC London Institute of Medical Sciences, Du Cane Road, London, W12 0NN UK

## Abstract

Translesion DNA synthesis is an essential process that helps resume DNA replication at forks stalled near bulky adducts on the DNA. Benzo[*a*]pyrene (B[*a*]P) is a polycyclic aromatic hydrocarbon (PAH) that can be metabolically activated to benzo[*a*]pyrene diol epoxide (BPDE), which then can react with DNA to form carcinogenic DNA adducts. Here, we have used single-molecule florescence resonance energy transfer (smFRET) experiments, classical molecular dynamics simulations, and nucleotide incorporation assays to investigate the mechanism by which the model Y-family polymerase, Dpo4, bypasses a (+)-*cis*-B[*a*]P-*N* ^2^-dG adduct in DNA. Our data show that when (+)-*cis*-B[*a*]P-*N* ^2^-dG is the templating base, the B[*a*]P moiety is in a non-solvent exposed conformation stacked within the DNA helix, where it effectively blocks nucleotide incorporation across the adduct by Dpo4. However, when the media contains a small amount of dimethyl sulfoxide (DMSO), the adduct is able to move to a solvent-exposed conformation, which enables error-prone DNA replication past the adduct. When the primer terminates across from the adduct position, the addition of DMSO leads to the formation of an insertion complex capable of accurate nucleotide incorporation.

## Introduction

**Figure 1:**
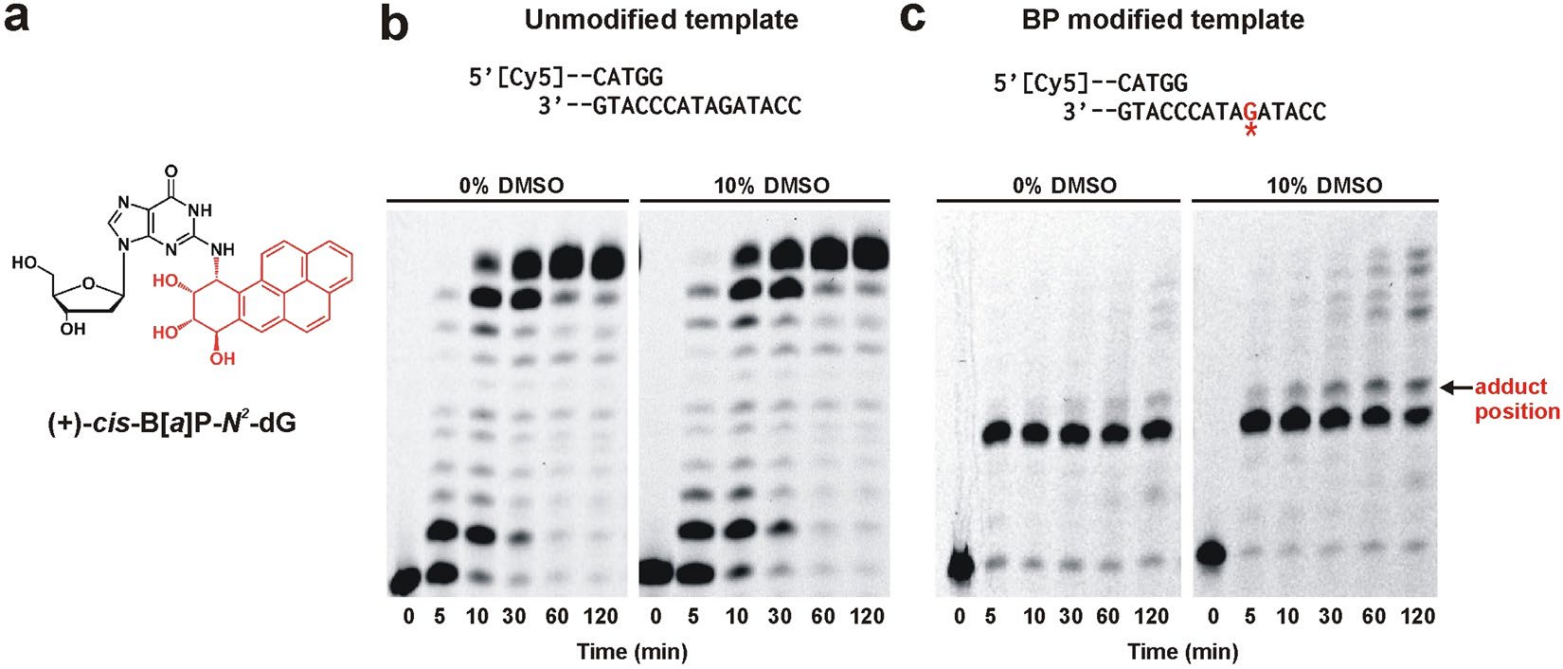
DMSO rescues stalled Dpo4 primer extension. (**a**) Chemical structure of the carcinogenic adduct (+)-*cis*-B[*a*]P-*N* ^2^-dG isomer. (**b**) Primer extension assay with unmodified template. DNA construct used for the assay is shown on top with 5′-Cy5 labelled primer (Table S1). Primer extension reaction was initiated with 2 nM Dpo4 and aliquots were quenched at the indicated time points before loading onto a denaturing acrylamide gel. The enzyme completes the extension in 30 min both in the absence and presence of DMSO. Both gel panels were taken from the same gel but separated to remove an intervening band. (**c**) Primer extension assay with B[*a*]P modified template. Position of the adduct is indicated by red G with an asterisk. Reaction was initiated with 50 nM Dpo4. The adduct stalls Dpo4 one nucleotide before the adduct position (arrow). 10% DMSO rescues stalled Dpo4 after 60 min. Both gel panels were taken from the same gel but separated to remove an intervening band.

Mutations in genomic DNA are caused by various environmental factors, such as radiation or certain chemicals and their metabolites^1^. Polycyclic aromatic hydrocarbons (PAHs) are a group of carcinogenic compounds that result from the incomplete combustion of organic materials. Sources of PAHs include automobile exhaust, powerhouses, refuse burning, smoked food and cigarette smoke^2–4^. The potent chemical carcinogen, benzo[*a*]pyrene (B[*a*]P) was first isolated in 1933 and is the most studied PAH^5^. B[*a*]P follows three major activation pathways that yield highly reactive metabolic products. These pathways include mechanisms involving the bay region dihydrodiol epoxide, one-electron oxidation, and dihydrodiol dehydrogenases pathways^6–9^. The primary consequences of these modifications are to increase B[*a*]P solubility and to facilitate excretion^10^. During these processes, B[*a*]P is converted to benzo[*a*]pyrene diol epoxide (BPDE) which reacts with *N* ^2^ of deoxyguanine (dG) to yield B[*a*]P-*N* ^2^-dG adducts in DNA, one of which being the (+)-*cis*-B[*a*]P-*N* ^2^-dG isomer (Fig. 1a)^7^. Structural studies have shown that the B[*a*]P adducts adopt multiple conformations in duplex DNA that lead to different effects on DNA replication^11^. Solution NMR studies of the (+)-*cis*-B[*a*]P-*N* ^2^-dG across from a complementary dC have revealed a displaced base intercalation orientation with the modified dG positioned in the minor groove and the pyrene ring moiety stacked in the DNA helix^12^.

B[*a*]P lesions are usually a strong block for high-fidelity polymerases and this leads to replisome stalling^13,14^. At stalled replication forks, damage-tolerant, translesion synthesis (TLS) allows DNA replication to resume^15^. TLS is essential for cell survival, and is performed by a specialized group of DNA polymerases called Y-family polymerases, which have been found to be present in all three domains of life^15^.

Both high fidelity and Y-family polymerases present a similar structural arrangement with a ‘palm’, ‘thumb’ and ‘fingers’ domain^16,17^. Contrary to high fidelity replicative polymerases, these Y-family polymerases are typically error-prone and lack 3′-5′ exonuclease activity^18^. The lesion bypass ability of Y-family polymerases is thought to be conferred by their unique structure, which presents a large, flexible and solvent-accessible active site that is able to accommodate bulky lesions during TLS^17,19^. In addition, Y-family polymerases contain a unique ‘little finger’ (LF) domain that largely determines the polymerase catalytic and bypass abilities^17,20,21^. Mammals have several Y-family polymerases, each one specialized for a different lesion type^22,23^.

DNA polymerase IV (Dpo4) from the thermophilic archaeon bacterium *Sulfolobus solfataricus* is a model Y-family polymerase homologous to human polymerase κ^17^. Extensive crystallographic studies have revealed that Dpo4 contains a spacious active site able to accommodate two templating bases simultaneously^17^. Also, because it has unusually small finger and thumb domains it has a very open active site with few DNA contacts leading to DNA slippage during synthesis and resulting in high levels of mismatch and frame-shift mutations^24,25^. In high fidelity DNA polymerases, correct dNTP incorporation is accomplished by a large conformational change in the thumb and fingers domain^26^, while crystal structures show that most Y-family polymerases, such as Dpo4, do not exhibit large conformational rearrangements in the ternary complex, which is also thought to affect the accuracy of nucleotide incorporation^17,27^.

Replication of B[*a*]P-modified DNA is highly error prone, and the error signature depends on the organism studied, the specific DNA sequence around the adduct position and the stereochemistry of the adduct^28–31^. Correlations of mutation spectra in lung tumors have directly implicated B[*a*]P adducts in cancer initiation^32,33^. It is well established that the mutagenic effects of B[*a*]P lesions depend on the adduct conformation in the DNA^34,35^. In some cases, more than one Y-family polymerase is required for successful primer extension across and after the adduct position^36,37^. It has been shown that the (+)-*cis*-B[*a*]P-*N* ^2^-dG adduct is highly mutagenic in simian kidney (COS7) and *E. coli* cells, and primarily causes G - > T transversions^35^. Once translesion synthesis has occurred, the bulky adduct can distort the DNA, activating the nucleotide excision repair (NER) pathway^38,39^.

In the present study, smFRET, MD simulations, and single nucleotide incorporation assays were used to characterize Dpo4 bypass of a DNA template containing (+)-*cis*-B[*a*]P-*N* ^2^-dG adduct. Our data show that Dpo4 binds to the B[*a*]P-modified DNA in two distinct conformations when the modified base is at the templating position. These conformations were identified as pre-insertion binary complex I and insertion binary complex I, each of which has the B[*a*]P adduct intercalated into the DNA helix, blocking nucleotide incorporation from occurring. However, Dpo4 can slowly extend the primer across from and after the adduct in the presence of DMSO by making a productive binary complex in which the (+)-*cis*-B[*a*]P-*N* ^2^-dG adduct is likely flipped out and exposed to the solvent. The smFRET experiments in the presence of DMSO reveal that Dpo4 also binds to form two complexes (insertion binary complex II and preinsertion binary complex II), and gel analysis shows that DMSO enhances the rate of incorporation across from the adduct. In the presence of DMSO, Dpo4 predominantly incorporates dG across from the adduct, producing a G:G mismatch. Finally, when the DNA primer terminates across the adduct, DMSO has a more prominent effect on the Dpo4 binding conformation because in its presence we observe a single FRET value. Accurate incorporation occurs at the position following the adduct for both the modified and unmodified templates.

## Results

### Stalled Dpo4 primer extension is rescued by DMSO

Successful DNA adduct bypass depends on various factors, such as the nature of the adduct, its orientation within the DNA helix, the flanking DNA sequence and the properties of the DNA polymerase. To investigate the blocking potential of the B[*a*]P adduct, we first performed running start primer extension assay by Dpo4 using either an unmodified or (+)-*cis*-B[*a*]P-*N* ^2^-dG-modified template (Fig. 1). The unmodified primer-template DNA construct results in efficient DNA synthesis, and the starting product is fully extended within 1 minute (Supplementary Fig. S1). Lowering the Dpo4 concentration reveals replication intermediates at early time points and full extension after 10 minutes (Fig. 1b, left panel). By contrast, the B[*a*]P-modified template results in Dpo4 stalling one base before the adduct (Fig. 1c). After 30 minutes of incubation, some incorporation across the adduct takes place, but fully extended product is not observed even after two hours (Fig. 1c).

Previous work using a (+)-*cis*-B[*a*]P-*N* ^2^-dA adduct has shown that the B[*a*]P aromatic moiety can adopt a stacked conformation within the DNA helix, similar to what is observed for the (+)-*cis*-B[*a*]P -*N* ^2^-dG adduct, effectively blocking replication by Dpo4^40^. In that study, it was shown that adduct stacking could be reduced by the addition of an organic solvent, such as DMSO, that reduced the dielectric constant of the media, presumably causing the adduct to adopt an alternate, more solvent exposed conformation and allowing increased adduct bypass^40^. Addition of DMSO not only provides a more hydrophobic environment that promotes the external positioning of the adduct, but it also more closely mimics the cellular medium^41^. To test whether a reduction in adduct stacking can also enhance bypass synthesis with the (+)-*cis*-B[*a*]P -*N* ^2^-dG adduct, we added 10% DMSO to the reaction buffer and measured DNA extension past the adduct. In agreement with the prior study, the presence of 10% DMSO allows Dpo4 to extend across and past the B[*a*]P adduct faster than in the absence of the organic solvent (Fig. 1c). Addition of 10% DMSO did not significantly alter DNA extension kinetics for the unmodified template (Fig. 1b). These data suggest that DMSO induces a conformational rearrangement of the B[*a*]P adduct that results in the formation of a more catalytically active complex.

### B[*a*]P adduct induces different Dpo4 binding conformations compared to unmodified DNA

**Figure 2:**
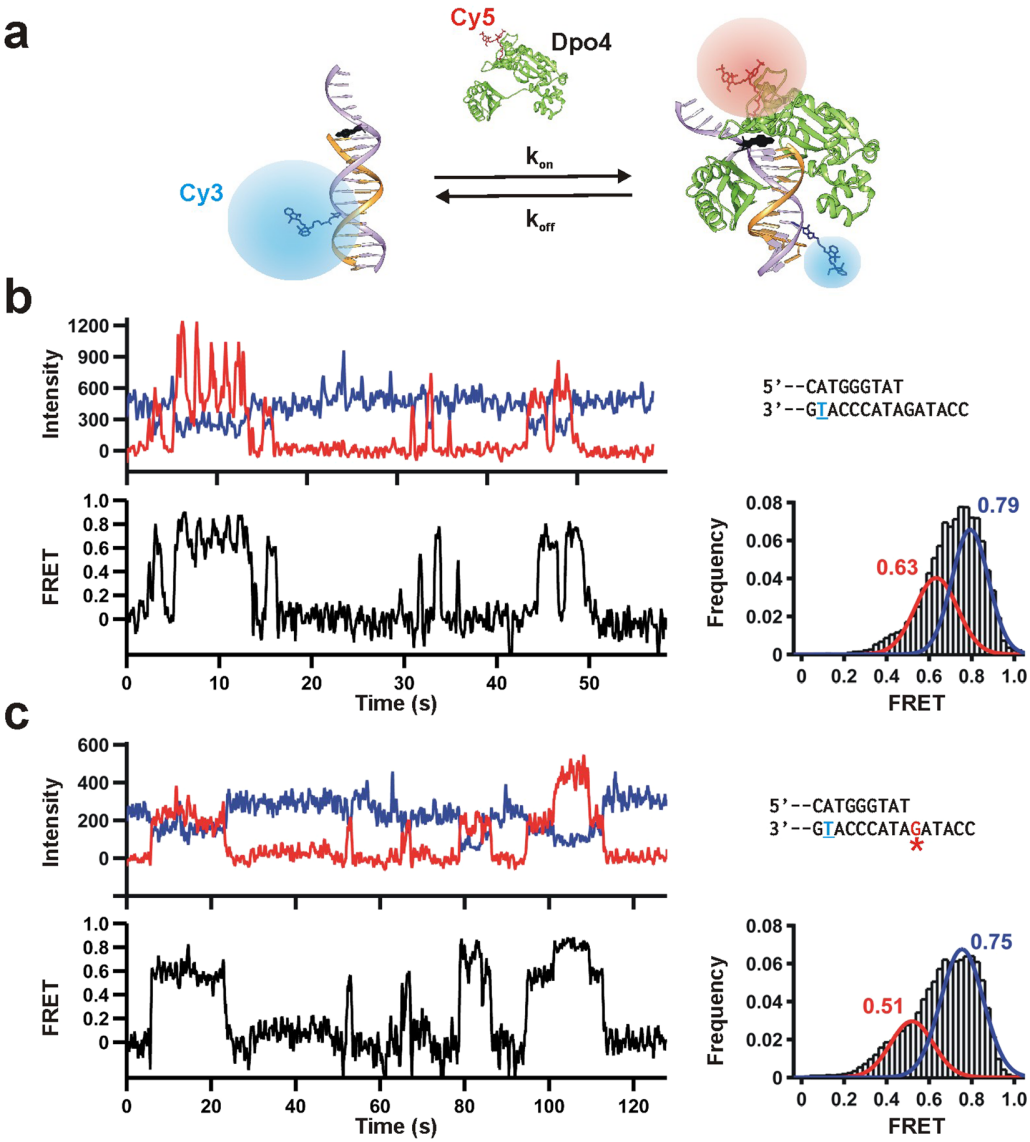
Single-molecule Dpo4 binding dynamics on unmodified and B[*a*]P-modified templates. (**a**) Schematic diagram of the smFRET setup to monitor the Dpo4 binding dynamics to DNA. Cy3-labeled primer-template is immobilized onto a quartz slide trough biotin-streptravidin and with Cy5-labeled Dpo4 in solution (The label is on the finger domain). Upon Dpo4 binding, FRET takes place between Cy3 and Cy5. (**b**) Characteristic smFRET Dpo4 binding trajectory to the unmodified DNA. DNA sequence is on the right (Table S1). Underlined T indicates Cy3 position. Dpo4 binding decreases Cy3 fluorescence (I_D_, blue) while Cy5-signal (I_A_, red) increases simultaneously. Apparent FRET efficiencies (bottom) are calculated as I_A_/ (I_A_ + I_D_). Time-binned smFRET histogram from >100 trajectories; bimodal distribution reveals two binding conformations. (**c**) smFRET Dpo4 binding trajectories to the B[*a*]P modified DNA. Red G indicates adduct location. Time-binned smFRET histogram from >100 trajectories yields a bimodal distribution revealing two binding conformations.

It has been shown that different Y-family polymerases display different efficiencies and fidelities for the same B[*a*]P adduct during TLS^42^. However, most of these ensemble-averaged experiments do not provide information about the polymerase binding dynamics in the population or sub-populations. Single-molecule techniques, such as smFRET, enable the monitoring of transient events and individual molecular populations^43^. To characterize the conformation and dynamics of Dpo4 on the adducted DNA using smFRET, we labeled the DNA with a FRET donor (Cy3) and the finger domain of Dpo4 with a FRET acceptor (Cy5, Fig. 2a). This labeling strategy has previously enabled us to characterize Dpo4 binding kinetics while monitoring the global conformation of the binary complex^44,45^. Unlike replicative polymerases, Y-family polymerases do not exhibit large conformational changes of the finger and thumb domains^46^, therefore, this labeling strategy only reports on the global conformation of the DNA-bound polymerase. The resulting single-molecule trajectories show free DNA as Cy3-only fluorescence, and Dpo4 binding as anti-correlated increases in Cy5 fluorescence and decreases in Cy3 (Fig. 2b). In agreement with our previous work^44^, the DNA-Dpo4 binary complex samples two FRET distributions for the unmodified DNA construct (Fig. 2b, right). The main population is centered at ~0.8 FRET, while the minor population is centered ~0.6 FRET. Based on our previous work^44^, we assign the high FRET peak (0.8) to a pre-insertion binary complex, and the low FRET peak (0.6) to the insertion binary complex. Single-molecule trajectories with the (+)-*cis*-B[*a*]P -*N* ^2^-dG-modified template also exhibit a similar behavior with two possible binding configurations (Fig. 2c). Analysis of ~100 single-molecule trajectories shows a bimodal FRET distribution, albeit with overall lower FRET values (peaks centered at ~0.5 and ~0.75, Fig. 2c, right), indicating that the (+)-*cis*-B[*a*]P-*N* ^2^-dG adduct slightly distorts the polymerase binding orientation on the DNA template.

### Dpo4 binds to B[*a*]P modified DNA in a catalytically active conformation in the presence of DMSO

**Figure 3:**
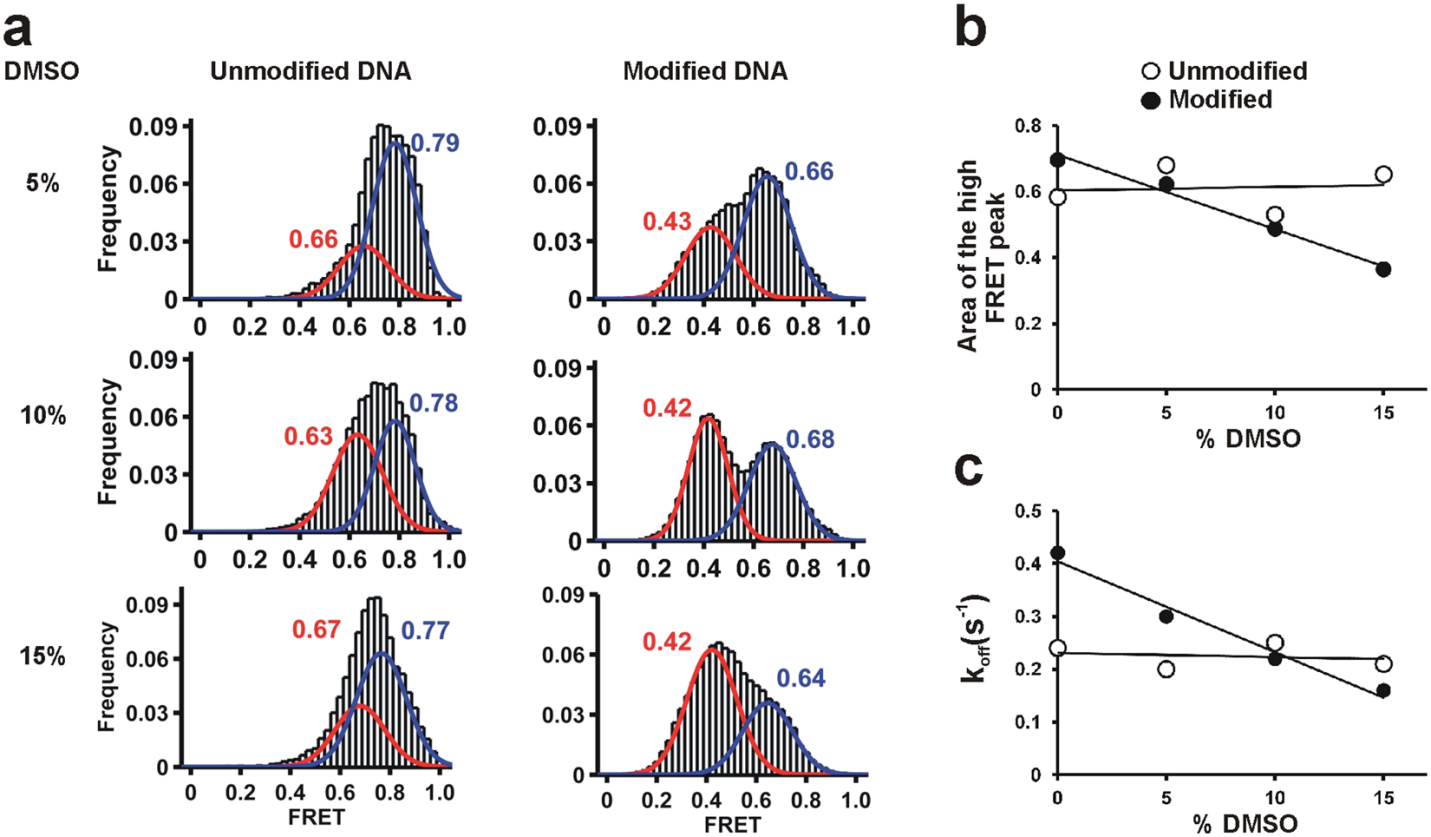
Dpo4 binding conformation of the (+)-*cis*-B[*a*]P-*N* ^2^-dG modified template. (**a**) smFRET histograms with increasing DMSO to monitor its effect on Dpo4 binding for the unmodified and modified DNAs. All histograms are fit to bimodal distributions. (**b**) Relative fraction area of high FRET state as a function of DMSO percentage in the buffer. Solid lines represent linear fits to the data. DMSO stabilizes the low FRET conformation for the adducted DNA but not the unmodified DNA. (**c**) Dpo4 dissociation rate constant (*k*_*off*_) as function of DMSO concentration. DMSO stabilized the modified binary complex but not with the unmodified DNA.

There is no structural information for the (+)-*cis*-B[*a*]P-*N* ^2^-dG adduct in complex with Dpo4. Early solution NMR studies of the (+)-*cis*-B[*a*]P-*N* ^2^-dG adduct in duplex DNA have shown that the B[*a*]P hydrocarbon moiety intercalates into the helix^12,47^. Crystal structures of the (+)-*cis*-B[*a*]P-*N* ^2^-dA DNA adduct in complex with Dpo4 show two possible conformations where B[*a*]P moiety is either stacked within the DNA helix or flipped out to the major groove^40^. The latter orientation is energetically unfavorable because the hydrophobic moiety is exposed to solvent, but presumably it can be stabilized in the presence of organic solvents, which have been shown to improve Dpo4 lesion bypass synthesis^40^. As shown above, the presence of DMSO also enhances Dpo4′s ability to bypass the (+)-*cis*-B[*a*]P-*N* ^2^-dG adduct. Therefore, we hypothesize that the (+)-*cis*-B[*a*]P-*N* ^2^-dG adduct behaves akin to the (+)-*cis*-B[*a*]P-*N* ^2^-dA adduct in the binary complex. To test this, we measured the binary complex orientation using smFRET in the presence of DMSO (Fig. 3a).

Addition of DMSO does not significantly affect the FRET distribution for the unmodified template, and the relative area under each peak remains essentially unchanged throughout the DMSO titration (Fig. 3b), suggesting that the equilibrium between the insertion and pre-insertion binary complexes is not altered by DMSO. In the presence of the B[*a*]P-modified template, increasing the DMSO concentration favors the low FRET conformation (designated insertion binary complex II) and shifts the FRET distribution towards lower values (Fig. 3b). We also measured the polymerase complex dissociation kinetics by analyzing the dwell-time distributions (Supplementary Fig. S2). For the unmodified template, Dpo4 dissociates with a rate constant *k*_*off*_ = 0.22 s^−1^, which agrees well with our previous data using a different DNA construct (0.20 s^−1^)^44^. This value remains unchanged in the presence of up to 15% DMSO (Fig. 3c). However, in the presence of the B[*a*]P-modified template Dpo4 dissociates approximately two-fold faster (*k*_*off*_ = 0.42 s^−1^), indicating that the adduct destabilizes the binary complex by about 0.32 kcal/mol. Addition of DMSO decreases *k*_*off*_ linearly (Fig. 3c) to 0.21, in agreement with the idea that the organic solvent favors the B[*a*]P solvent-exposed conformation, thus re-stabilizing the binary complex.

### Dpo4 favors dG misincorporation across (+)-*cis*-B[*a*]P-*N*^2^-dG adduct

Y-family polymerases do not have proofreading activity, which is an inherent property of high fidelity polymerases. Therefore, Y-family polymerases have higher error rates (in the 10^−2^–10^−3^ range) when copying normal DNA^48^. To study the preference of Dpo4 nucleotide incorporation in the ternary complex, we first used a single-nucleotide incorporation assay with unmodified DNA (Supplementary Fig. S3). Dpo4 mainly incorporates the next correct nucleotide, dC, across from the dG in the unmodified template in the presence or absence of DMSO. However, other non-complementary bases can also be incorporated depending on the Dpo4 concentration and reaction time (Supplementary Fig. S3b).

**Figure 4:**
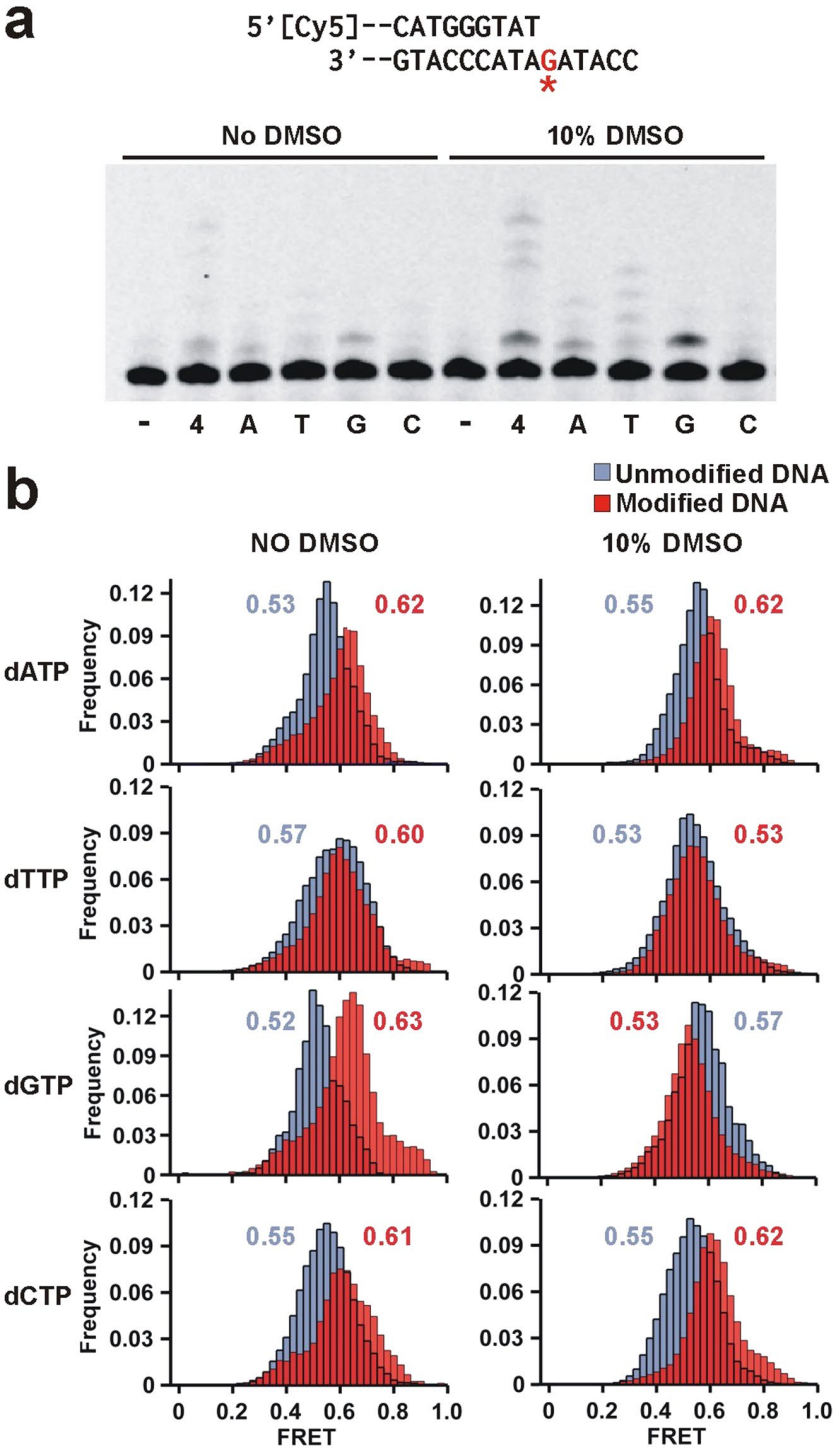
Dpo4 binding dynamics to the DNA with adducted dG as templating base in the presence of each nucleotide. (**a**) Modified DNA sequence used for this single nucleotide incorporation assay (top, Table S1). The label ‘-‘ indicates no nucleotide added and ‘4’ the presence of all four nucleotides. The other lanes contain only the specified nucleotide. Nucleotide incorporation is minimal in the absence of DMSO. In the presence of DMSO dG is incorporated across the adduct. (**b**) smFRET distributions for Dpo4 binding to unmodified (blue) and B[*a*]P modified (red) DNA in the presence of 1 mM dNTP as indicated, in the absence (left) and presence (right) of 10% DMSO. When nucleotides are present all histograms fit to single Gaussian distribution.

We next carried out single-nucleotide incorporation assays to characterize the Dpo4 error signature across ( + )-*cis*-B[*a*]P-*N* ^2^-dG. In the absence of DMSO, DNA extension proceeds inefficiently with minor amounts of dG and dA mostly being incorporated (Fig. 4a). The same reaction in 10% DMSO yields ~24% incorporation, with dG being preferentially incorporated across the adduct (Fig. 4a), thus resulting in the formation of a G:G mismatch. These results suggest that the DMSO-induced solvent exposed conformation of (+)-*cis*-B[*a*]P-*N* ^2^-dG adduct is more catalytically active but does not adopt a conformation permitting correct dNTP incorporation.

To investigate the possible ternary complex conformations leading to this error signature, we carried out smFRET experiments in the presence of each nucleotide. To prevent dNTP incorporation, we used Ca^2+^ as the divalent metal ion instead of Mg^2+.^, a substitution that has been used in the past for inhibiting nucleotide incorporation by Dpo4^44,49^. In agreement with our previous results, binding to an unmodified template in the presence of each of the four dNTPs produces a single low FRET distribution (Fig. 4b, left panels, blue), a change we attribute to be the formation of the insertion complex. When the template contains a (+)-*cis*-B[*a*]P-*N* ^2^-dG adduct and in the presence of each dNTP, the (red) FRET distributions now have a higher value (~0.60), indicative of the formation the pre-insertion complex (Fig. 2a) and suggesting that the adduct precludes formation of the insertion conformation, thereby preventing DNA synthesis.

Addition of 10% DMSO does not significantly alter the peak positions of the ternary complex with unmodified templates (Fig. 4b, right panels, blue), except for slight shifts observed for dGTP and dTTP. With the modified templates and in the presence of either dATP or dCTP, we observe a higher (red) FRET distribution (~0.62), indicative of the formation the pre-insertion complex and consistent with the observation that these two nucleotides are not incorporated (Fig. 4a). The only distribution that displays a decrease in the presence of a nucleotide and DMSO is the dGTP ternary complex, where a FRET value (~0.53) for the modified template is observed that presumably corresponds to the formation of the catalytically active insertion complex and is consistent with the observation that dGTP is the only nucleotide incorporated (Fig. 4a). Interestingly, the FRET distribution (~0.53) observed for the dTTP ternary complex is the same for the unmodified and modified templates. It is possible that with the modified template and in the presence of DMSO, the dTTP ternary complex can adopt an inactive conformation that resembles the insertion complex, possibly caused by bulging out of the adducted base which would permit base pairing with the next template base (A).

### B[*a*]P adduct at duplex DNA terminus is bypassed in an error-free manner

**Figure 5:**
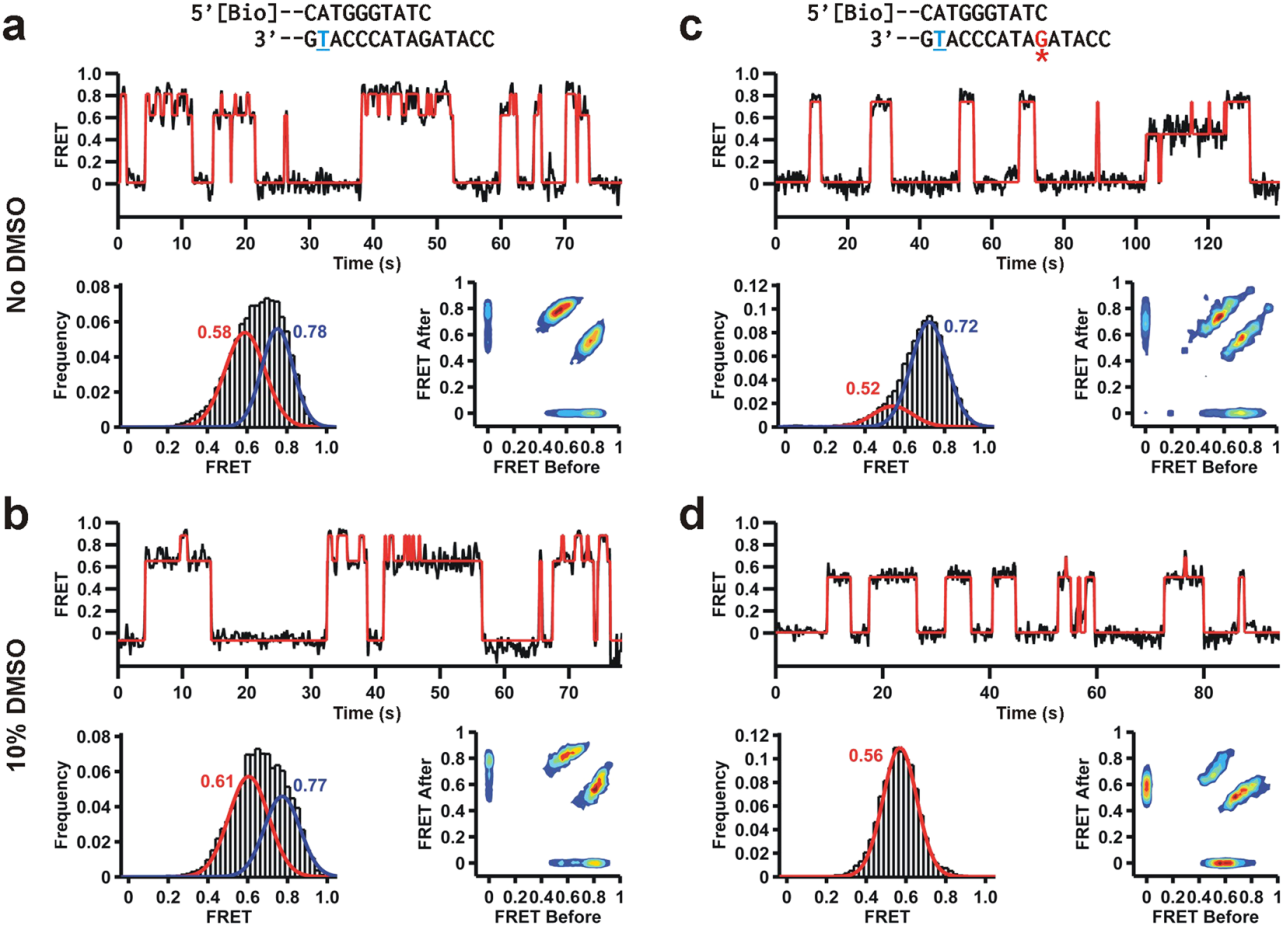
Dpo4 binding dynamics to the DNA where the primer terminates across the adduct. Red line shows HHM fit. (**a**) Dpo4 binding to the unmodified DNA in the absence of DMSO. Characteristic FRET trajectory (top), histogram (bottom left) with bimodal fit and transition density plot (TDP, bottom right). (**b**) Dpo4 binding to the unmodified DNA in the presence of 10% DMSO. Characteristic FRET trajectory, and resulting histogram and transition density plot (TDP). (**c**) Dpo4 binging to the adducted DNA in the absence of DMSO. Characteristic FRET trajectory, and resulting FRET histogram and TDP. (**d**) Dpo4 binging to the adducted DNA in the presence of 10% DMSO. Characteristic FRET trace, FRET histogram and TDP.

We next determined how a (+)-*cis*-B[*a*]P-*N* ^2^-dG adduct bound to the terminal base pair of the primer-template affects the binary complex conformation. As expected for the unmodified primer-template in the absence of DMSO, Dpo4 samples the two conformations that we have previously assigned to the pre-insertion (FRET ~0.8) and insertion binary complexes (FRET ~0.6, Fig. 5a). In the presence of 10% DMSO, the FRET distribution does not significantly change and still exhibits two main populations, showing again that the organic solvent does not alter the equilibrium between the insertion and pre-insertion binary complexes (Fig. 5b). Symmetric transition density plots at both 0% and 10% DMSO show reversible shuttling between the insertion and pre-insertion conformations (Fig. 5a and b).

When the B[*a*]P adduct is present at the DNA duplex terminus and in the absence of DMSO, the FRET distribution solution shows two peaks centered near ~0.7 and ~0.5, presumably corresponding to slightly perturbed conformations of the pre-insertion complex I and insertion binary complex I as observed in Fig. 3 (Fig. 5c). Addition of 10% DMSO results in a dramatic change of the FRET distribution that only exhibits a single peak centered ~0.55. This result shows that in 10% DMSO Dpo4 samples only the B[*a*]P solvent-exposed conformation, which results in a global conformation akin to the insertion binary complex II (Fig. 5d).

**Figure 6:**
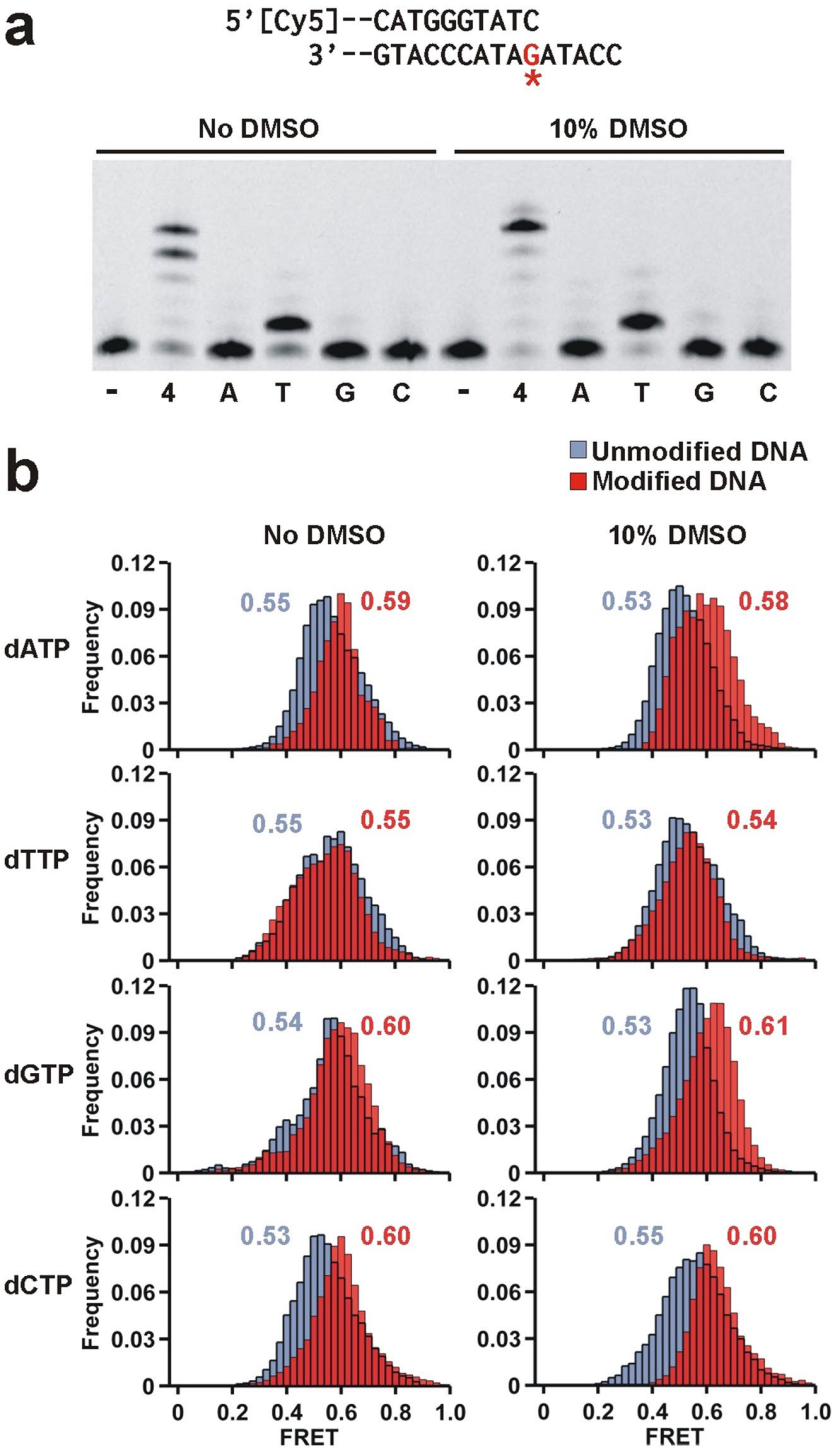
Dpo4 incorporates the correct dNTP after the adduct position. (**a**) Single nucleotide primer extension of the adducted DNA in the absence and presence of DMSO. The label ‘-‘ indicates no nucleotide added and ‘4’ the presence of all four nucleotides. The other lanes contain only the specified nucleotide. After the adduct position there is no observable difference in Dpo4 synthesis with DMSO. (**b**) smFRET distributions for Dpo4 binding to unmodified (blue) and B[*a*]P modified (red) DNA in the presence of 1 mM dNTP as indicated, in the absence (left) and presence (right) of 10% DMSO. When nucleotides are present all histograms fit to single Gaussian distribution.

Extension of DNA with (+)-*cis*-B[*a*]P-*N* ^2^-dG adduct at the duplex terminus by Dpo4 takes place mostly error-free in this sequence context (Fig. 6a), as dT is the only nucleotide showing significant incorporation across from dA. In the presence of 10% DMSO, the degree of dNTP incorporation does not change significantly, indicating that the adduct adopts the same conformation in the presence or absence of DMSO. The same DNA primer with unmodified template shows similar dT incorporation across the dA (Supplementary Fig. S4). In agreement with these results, the FRET distribution in the presence of the next correct nucleotide dT is virtually identical in 0% and 10% DMSO (Fig. 6b). Moreover, this FRET value (0.55) matches the one observed for the unmodified DNA construct in the presence of dT, suggesting that the conformation of the cognate ternary complexes is very similar for unmodified and B[*a*]P modified DNA constructs.

Addition of incorrect nucleotides dA, dC and dG for the B[*a*]P-modified DNA results in higher FRET values (~0.6) when compared to the cognate dT (Fig. 6b). The tertiary complex binding conformation only resembles the ones observed with the unmodified DNA in the presence of the correct dTTP. As we have previously shown, for unmodified DNA all four dNTPs result in similar FRET distributions (~0.54), suggesting that cognate and non-cognate ternary complexes present an overall similar structural arrangement.

### Misincorporated dG forms a stable conformation across the ( + )-*cis*-B[*a*]P-*N*^2^-dG adduct

**Figure 7:**
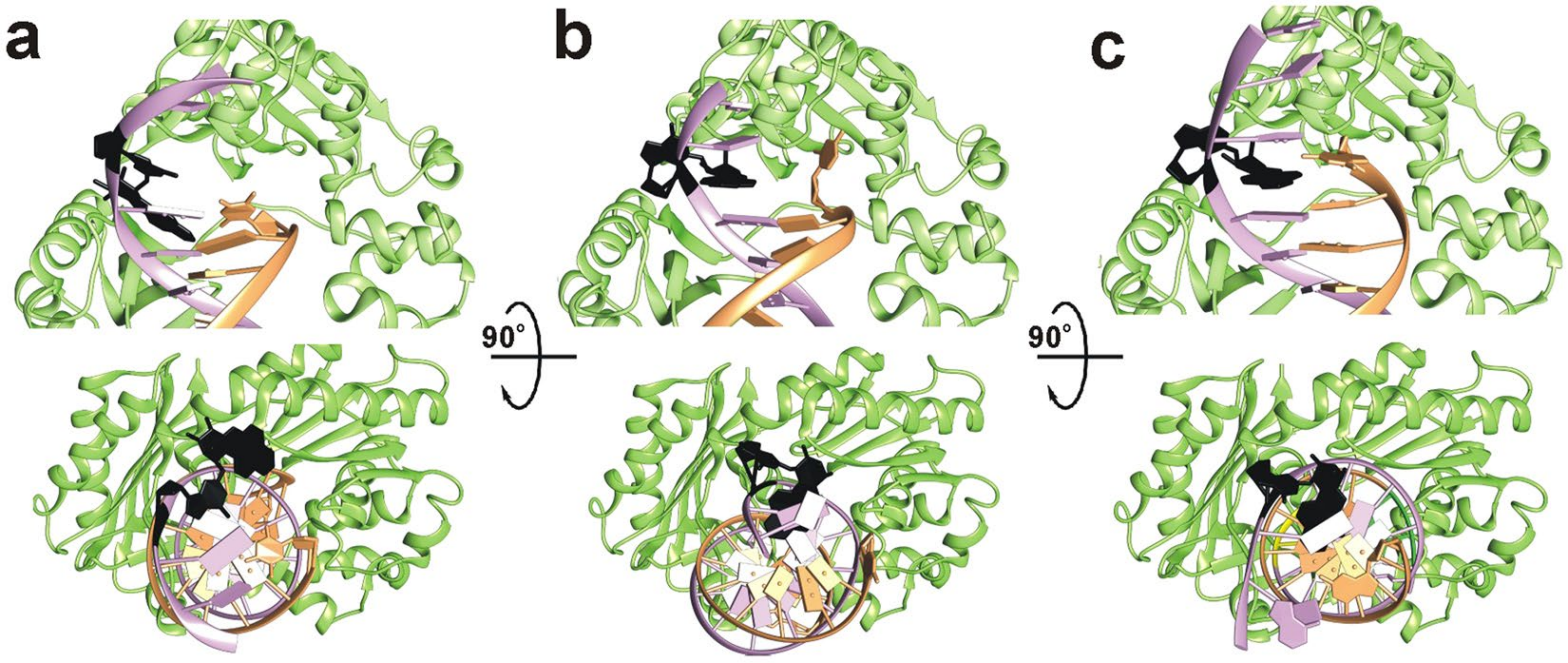
Representative structures of the DNA helix from the end of three MD simulations. (**a**) The structure of B[a]PG without a corresponding nucleobase at the end of 100 ns of MD in a pure water system. (**b**) The structure of B[*a*]PG-dC at the end of 100 ns of MD in a pure water system. **(c)** The structure of B[*a*]PG-dG at the end of 100 ns of MD in a pure water system.

Incorporation of dG across the (+)-*cis*-B[*a*]P-*N* ^2^-dG adduct by Dpo4 is strongly supported by biochemical and single molecule data (Fig. 4). To gain further insights, we performed several molecular dynamics simulations of various systems. All simulations have RMSDs below 2.5 Å on average, indicating that the protein structures were fairly stable in the simulations. Further details of the RMSD results and calculations can be found in the supporting information (Supplementary Fig. S5). To confirm the stability of the (+)-*cis*-B[*a*]P-*N* ^2^-dG:dG (B[*a*]P-dG:dG) mismatch, and to show that the force field parameters for the computational simulations were consistent with those in the literature, we investigated the relative structure of the DNA in three similar systems with different nucleotides across from the adduct (Fig. 7). Figure 7a denotes a structure where the (+)-*cis*-B[*a*]P-*N* ^2^-dG has no corresponding nucleobase, i.e. the binary preinsertion complex. In this case, the adduct over the course of the simulation becomes stacked within the minor groove and distorts the attached nucleobase and backbone structure substantially. Structures in Fig. 7b and c are consistent with previous DNA-only calculations by Mu *et al*. with the adduct located in the mid-DNA chain rather than at the primer-template junction^38^. Our (+)-*cis*-B[*a*]P-*N* ^2^-dG:dC (B[*a*]P-dG:dC) calculations show that the adduct remains stacked within the major groove and the adducted G base remains stacked within the minor groove for the duration of the simulation (Fig. 7b), consistent with the DNA-only calculations but with a missing hydrogen bond, likely due to the location of the dC base at the end of the primer. The (B[*a*]P-dG:dG) calculations (Fig. 7c) show that the adduct remains stacked within the major groove, the adducted dG base is stacked within the minor groove, and the corresponding dG base orientation is stacked within the major groove and on top of the adduct, also consistent with our experimental results and the DNA-only calculations^38^.

**Figure 8:**
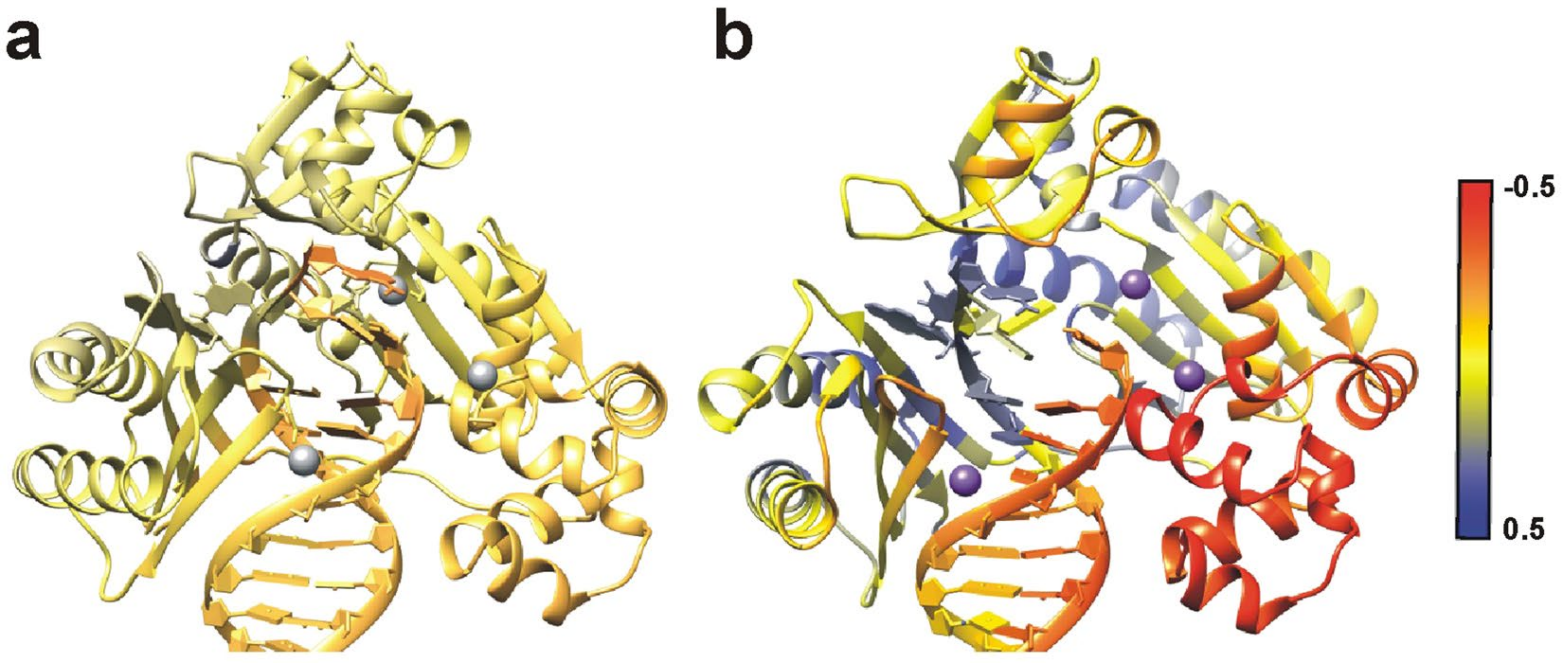
Difference correlation analysis relative to the adducted B[a]P-dG residue. Red denotes an increase in anticorrelation, yellow denotes no difference in correlation, and blue denotes an increase in correlation. The color scale has been rescaled to a range of −0.5 to 0.5 for clarity. (**a**) The difference in correlation between B[*a*]P-dG:dC in pure water and B[*a*]P-dG:dG in pure water relative to the adducted residue with the calcium ions colored in gray. (**b**) The difference in correlation between B[*a*]P-dG:dC in 10% DMSO/90% water with a solvent-exposed adduct conformation and B[*a*]P-dG:dC in pure water with the calcium ions colored in purple.

In addition, to further investigate the motions and structure of the protein, several sets of dynamic structural analyses were performed. Cross-correlation analysis was done on the B[*a*]P-dG:dC and B[*a*]P-dG:dG systems in water and 10% DMSO to identify differences in the relative movements of the residues in each trajectory. Additionally, two different sets of residue-wise correlation analysis subtractions (difference correlation) were performed—first, between two systems in water with differing nucleotides across from the adducted base (dC and dG) and second between one system in DMSO with a solvent-exposed conformation and one system in water, with the same nucleotide across from the adducted base (dC). Finally, the difference correlation associated with the adducted base was extracted and mapped onto the protein and DNA (Fig. 8). The difference in correlation between the two systems in pure water is fairly negligible, indicating that the movement of the protein and DNA residues relative to the adduct is essentially the same regardless of the corresponding nucleotide, despite the differences in DNA conformation (Fig. 7). In DMSO, however, there are significant differences between the correlation relative to the adducted base, especially for the section of protein around the adducted base and near the active site. The correlated movement of the palm domain of Dpo4 relative to the adducted base in its flipped-out orientation is reasonable considering the change in orientation. Also interesting is the increase in anti-correlated movement of the thumb region of Dpo4, since that particular region is associated with the translocation of the DNA. We also measured the distance between the two tagged sites in the FRET experiments over the course of the MD simulations. Compared to the pure water simulations, the trajectories solvated with 10% DMSO show a slight distance increase of 2 Å on average after equilibration, and up to 6 Å at various points. The trajectories show when the adduct is flipped out the finger domain is pushed up slightly, increasing the distance between the tagged sites, consistent with the observed FRET decrease in the single molecule data (Figs 2 and 3). Note that this is only a qualitative comparison given the difference in time-scales between the MD simulations and FRET experiments.

The large-scale motions of the protein were also explored with principal coordinate analysis (PCA), which shows substantial differences in the largest movements of the protein between the (B[*a*]P-dG:dC) system in water versus in 10% DMSO. The details of this analysis can be found in the supporting information.

**Figure 9:**
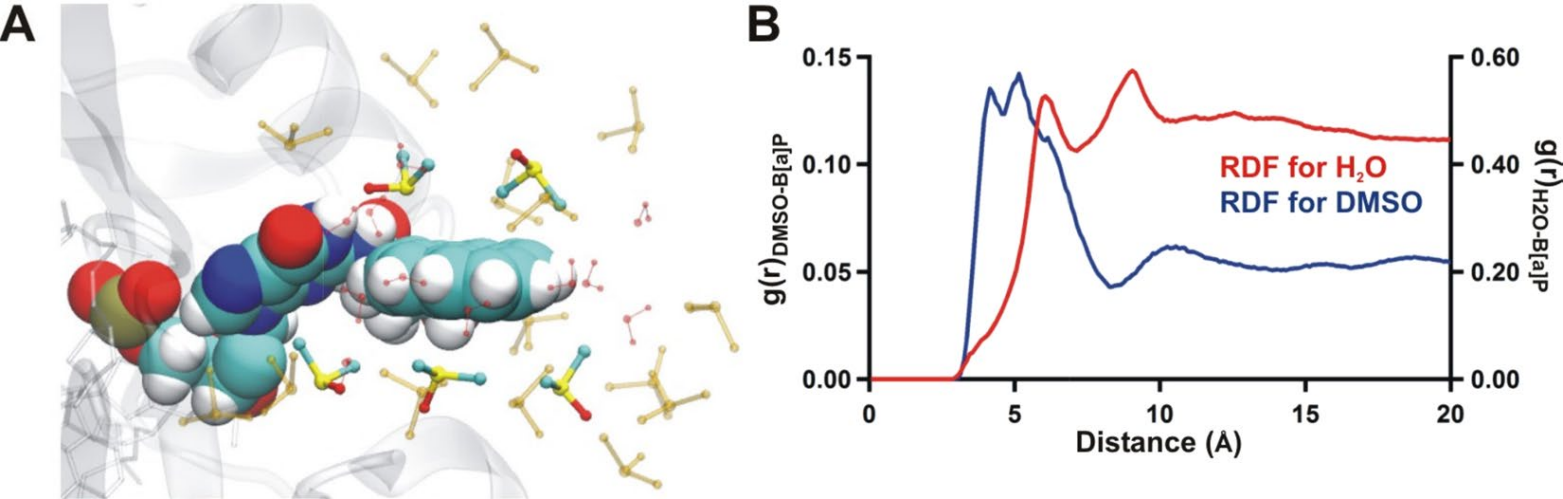
(**a**) Representative structure of the solvent-exposed adduct in 10% DMSO/90% water after 100 ns of MD simulation. The adduct is represented in VDW, the first solvation shell of DMSO (within 5.15 Å) is represented in ball and stick, the second solvation shell of DMSO (within 10.15 Å) is represented by ball and stick and colored transparent yellow, and the first solvation shell of water (within 5.15 Å) is represented by ball and stick and colored transparent red. (**b**) Calculated average radial distribution function over the entire 100 ns MD trajectory between the C3B atom in the B[*a*]P adduct and the S atom in the DMSO solvent molecules, and between the C3B atom in the B[*a*]P adduct and the O atom in water.

To investigate the effect of solvation in 10% DMSO on the adduct, a radial distribution function (RDF) for the C3B atom in the adduct (near the middle of the B[*a*]P adduct, with the same atom naming scheme as in PDB 2IA6) and the sulfur atoms in the DMSO solvent was calculated (Fig. 9b). The DMSO RDF adopts two distinct conformations, with either the sulfur or the methyl groups facing the B[*a*]P rings, which in this case can be seen most clearly above the adduct (Fig. 9a). On average, it is observed that when the sulfur from DMSO is pointing towards the B[*a*]P the distance between this atom and C3B of B[*a*]P is around 4.5-5 Å. Conversely, when the methyl groups of DMSO are pointing towards the B[*a*]P the distance is increased to 5-5.5 Å. This helps explain the splitting observed in the first peak, since the different conformations result in slightly different distances between the two molecules.

There are two distinct peaks at 5.15 and 10.15 Å, indicating two solvation shells of DMSO around the adducted base. Figure 9a shows a representative structure with for the solvation around B[*a*]P. The colored and solid DMSO molecules are within 5.15 Å of the adduct, whereas the transparent yellow DMSO molecules are within 10.15 Å of the adduct. Additionally, all water molecules within 5.15 Å from the adduct are displayed in transparent red. Our results indicate that there are no water molecules interacting with the top or bottom of the adduct; only a few along the equatorial hydrogens on the side of the ring structure, which is represented in the RDF of water as the first maxima. Taken together, these results suggest that the adduct is microsolvated by DMSO. Overall, our simulation results are consistent with the smFRET results presented above, which indicate that the solubility of the B[*a*]P is increased in 10% DMSO and therefore that a solvent-exposed conformation would be much more favorable.

## Discussion

Exposure to exogenous mutagens is one of the main causes of somatic mutations, some of which eventually lead to cancer. One means for understanding the mutagenic outcome of DNA damage is to visualize how DNA polymerases interact with lesions formed by various carcinogenic DNA adducts. B[*a*]P shows complicated carcinogenic properties because it forms different isomeric DNA adducts that each shows distinct mutational patterns in DNA. Most research about B[*a*]P carcinogenesis has focused on the more abundant (+)-*trans*-B[*a*]P but we believe that a complete understanding of the mutagenic properties of B[*a*]P requires an in-depth characterization of all isomers resulted from BPDE reaction with DNA bases. Therefore, in this work we have used smFRET, primer extension assays and molecular dynamics simulations to study the mechanistic implications of bypass of a bulky lesion, (+)-*cis*-B[*a*]P-*N* ^2^-dG by the model Y-family polymerase Dpo4.

Even though Y-family polymerases are essential for TLS of bulky B[*a*]P-DNA adducts, it is not known if a single Y-family polymerase can bypass all the B[*a*]P stereoisomers^42^. Y-family polymerases have been shown to incorporate both correct and incorrect dNTPs across the B[*a*]P adduct. The ability to successfully synthesize past the adduct also depends on the nature of the lesion^42^. Many studies have shown that primer extension after the adduct is severely inhibited or slowly rescued and may require the action of a second polymerase^42,50,51^. For example, Frank *et al*. have found evidence suggesting two Y-family polymerases are required for successful B[*a*]P-dA bypass: Pol ι incorporates mostly dT across a B[*a*]P-dA adduct and Pol κ extends past the terminal B[*a*]P-dA:dT base pair^37^.

Although the precise mechanism by which bulky adducts inhibit high-fidelity DNA polymerases is not known, there is evidence that these structures may prevent nucleotide incorporation by precluding the large finger-closing movement required for dNTP incorporation^52,53^. Y-family polymerases do not generally undergo such large rearrangements during their catalytic cycle and also present wider active sites that can presumably accommodate a bulky adduct structure. Therefore, it is believed that inhibition of Y-family polymerases activity by adducted DNA bases occurs through major alteration of the cognate DNA/Pol complex structure^25^. The most important and non-mutually exclusive distortion mechanisms include stacking of an aromatic moiety within the DNA helix, flipping the adducted base to a *syn* conformation and misalignment/rotation of the primer-template DNA strands. Even though Dpo4 is a versatile polymerase able to bypass a wide variety of DNA damage, the strong roadblock posed by (+)-*cis*-B[*a*]P-*N* ^2^-dG suggests that the DNA/Pol structure is severely distorted by the adduct.

**Figure 10:**
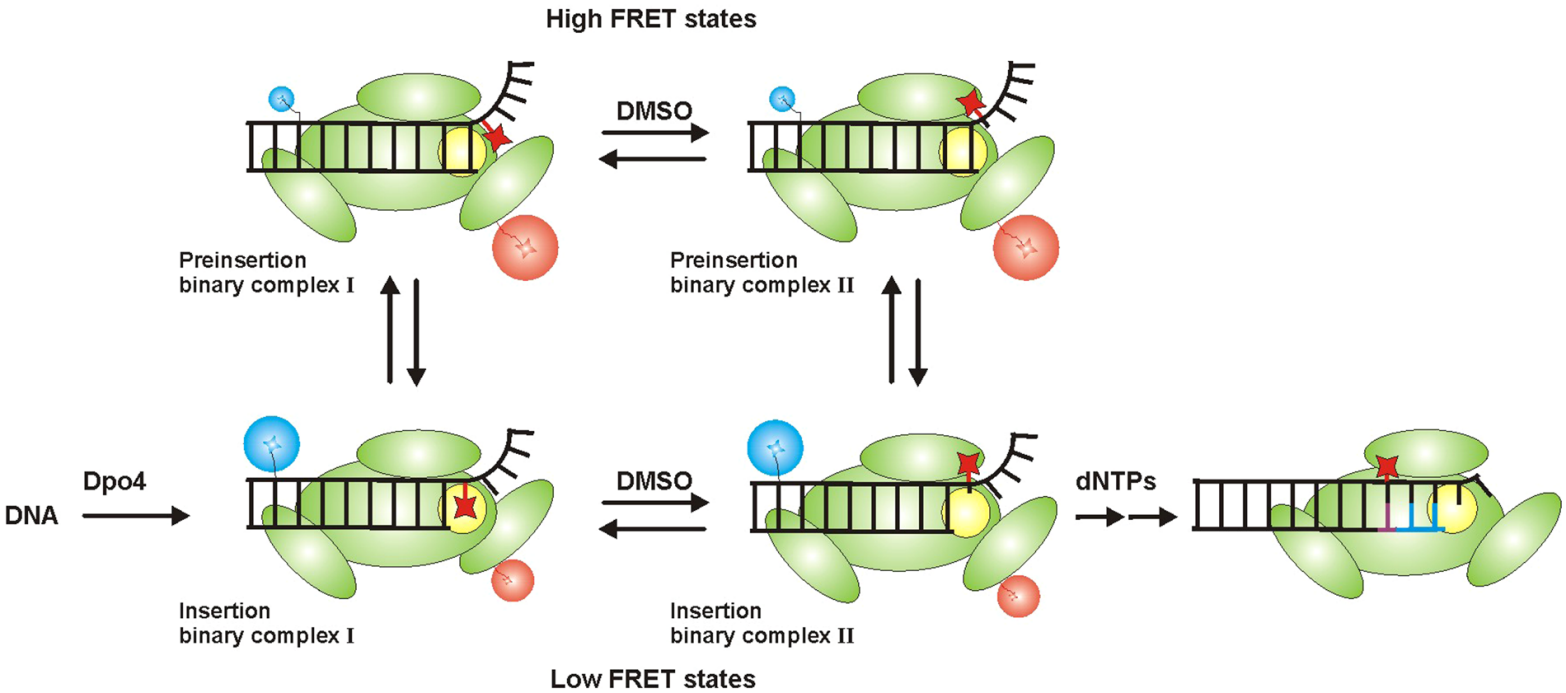
Proposed model shows different Dpo4 binding conformations on (+)-*cis*-B[*a*]P-*N* ^2^-dG (red star) modified DNA and possible mechanistic implications that permit Dpo4 for bypassing the adduct. When Cy3 (blue) labelled DNA binds to Cy5 (red) labelled Dpo4 (green), it forms the insertion binary complex I where the adduct locates Dpo4 active site (yellow) in the intercalated orientation. The adduct bypassing is prohibited in this conformation. At the same time, Dpo4 samples high FRET state, preinsertion binary complex I where the terminal base of the primer is located in the Dpo4 active site which is again unfavorable conformation for primer extension. We found that in the presence of DMSO, Dpo4 forms a binding conformation which permits Dpo4 to bypass the adduct forming insertion binary complex II. The adduct is in the exposed conformation and Dpo4 active site is opened for dNTP incorporation. Even with DMSO, Dpo4 is equilibrated between two FRET states forming another high FRET conformation, preinsertion complex II. In the absence of DMSO, preinsertion binary complex I generates a higher population whereas insertion binary complex II forms a higher population with DMSO. Formation of insertion binary complex II can follow either pathway. Upon addition of dNTPs, the primer extension can occur from insertion binary complex II. The G:G mismatch bond is shown in red line and newly formed complementary bases are shown in blue lines.

The structures generated by MD simulations have revealed the stacked and distorted adduct conformations at the primer template junction (Fig. 7), as discussed in the results section. Similar to other B[*a*]P adducts, the longer pause occurs during dNTP incorporation opposite the damaged base. No crystal structures exist with a (+)-*cis*-B[*a*]P-*N* ^2^-dG adduct as the templating base, but our data suggests that the aromatic moiety of B[*a*]P is present in one of two conformations: either stacked within the DNA helix or in a solvent-exposed, catalytically active conformation (Supplementary Fig. S6). Interestingly, even though different B[*a*]P isomers show distinct error signatures, the DNA synthesis-blocking mechanism seems to share similar features among all B[*a*]P adducts and it implies some degree of B[*a*]P stacking in the DNA helix (Fig. 10).

One remarkable feature of Dpo4 synthesis is that it incorporates mostly dG across from a (+)-*cis*-B[*a*]P-*N* ^2^-dG adduct, a quite uncommon base insertion, and does not show any tendency to incorporate the correct nucleotide dC. When the dC base is placed opposite B[*a*]P adduct, it flips out from the helix, showing an unfavorable interaction with the adducted base (Fig. 7b). This indicates that, although the (+)-*cis*-B[*a*]P-*N* ^2^-dG adduct is a minor reaction product, it has the potential to be a strong mutagen with an unusual mutational signature. Moreover, the fact that this mutation was not observed in *E. coli* or COS7 cells^35^ suggests that pol κ (the eukaryotic Dpo4 homologue) may not be responsible for synthesis across from the (+)-*cis*-B[*a*]P-*N* ^2^-dG adduct. Also, the fact that synthesis past the adduct is accurate may imply that this polymerase takes on that synthetic role. Unlike what is observed using the (+)-*trans*-B[*a*]P-*N* ^2^-dG adduct, Dpo4 does not use the downstream templating base for damage bypass with the *cis* adduct. This suggests that, for the (+)-*cis*-B[*a*]P-*N* ^2^-dG adduct, a misaligned structure with the adducted base looped-out (and not base-paired) is catalytically inefficient or not favored. Therefore, in this case we conclude that nucleotide incorporation takes place with the adducted dG sitting in the active site, and adopting alternate rotameric and/or tautomeric conformations to enable dG:dG pairing. In agreement with this idea, recent molecular dynamics simulations have shown that dG is the most stable nucleotide that can pair with a (+)-*cis*-B[*a*]P-*N* ^2^-dG adduct^38^. It appears that the dG:dG base pair is stabilized by some of the major groove contacts of the purine ring system. Also, the least nucleotide excision repair efficiency is observed when dG is the partner of the (+)-*cis*-B[*a*]P-*N* ^2^-dG adduct suggesting that this mispaired structure is the least distorting.

When the primer is extended one nucleotide and the (+)-*cis*-B[*a*]P-*N* ^2^-dG adduct sits at the primer-template junction, our data is also consistent with a (+)-*cis*-B[*a*]P-*N* ^2^-dG stacked solvent–exposed conformation, which is presumably more catalytically active. In contrast with our prior studies with the aromatic amine-dG adducts, Dpo4 extends past the (+)-*cis*-B[*a*]P-*N* ^2^-dG adduct in an error-free manner, suggesting that the solvent-exposed conformation does not dramatically distort DNA at the Dpo4 active site.

Shown in Fig. 10 is a model that incorporates the various binding conformations we observe into a possible mechanistic pathway that allows Dpo4 to bypass the (+)-*cis*-B[*a*]P-*N* ^2^-dG adduct. When Dpo4 binds to the (+)-*cis*-B[*a*]P-*N* ^2^-dG-modified DNA, we observe two distinct Dpo4 binding conformations that are in dynamic equilibrium (insertion binary complex I and preinsertion binary complex I, Fig. 10). Dpo4 prefers to form the preinsertion complex I, where the terminal base pair occupies the dNTP binding site^44^, but we also observe a substantial fraction of the complex in the insertion I conformation. This latter structure places the (+)-*cis*-B[*a*]P-*N* ^2^-dG in the templating position so that the B[*a*]P is in the active site blocking incorporation (Fig. 10), as evidenced by our primer extension results (Fig. 1).

In the presence of DMSO, smFRET experiments provide evidence for the existence of two new binding conformations that we label preinsertion binary complex II and insertion binary complex II both of which presumably have the (+)-*cis*-B[*a*]P-*N* ^2^-dG adduct flipped out in a solvent exposed orientation (Fig. 10). These two conformations are also in equilibrium, with insertion binary complex II being most stable. Because this structure has neither the terminal nucleotide nor the B[*a*]P adduct in the active site, this complex is able to incorporate a nucleotide across from the modified base, but because the adduct alters the templating base orientation, incorporation is highly inaccurate. MD simulations for the binary complex in the presence of 10% DMSO also shows the formation of the solvent exposed conformation of the (+)-*cis*-B[*a*]P-*N* ^2^-dG adduct in the primer-template junction (Supplementary Fig. S6). Furthermore, the solvent exposed B[*a*]P adduct seems to be sandwiched between the LF and finger domain of the Dpo4 (Supplementary Fig. S6). Using the same model shown in Fig. 10 to interpret the analogous Dpo4 binding conformations for the DNA construct where the (+)-*cis*-B[*a*]P-*N* ^2^-dG adduct is located at the primer terminus with a dC partner base, we observe both insertion and preinsertion complex I, with the preinsertion complex predominating. The addition of DMSO leads to the predominant formation of insertion binary complex II, presumably because the B[*a*]P adduct is preventing translocation to the preinsertion complex.

In summary, we were able to identify the distinct binding conformations between Dpo4 and (+)-*cis*-B[*a*]P-modified DNA in real-time using smFRET. The MD simulations provide confirmation of the biochemical and smFRET results. Although primer extension is strongly inhibited by stacking of the B[*a*]P moiety into the DNA helix, the presence of DMSO generated a structure that allowed error-prone incorporation across from the adduct position. Our results provide a mechanistic basis for the stepwise process by which a Y-family polymerase extends across and past a bulky adduct in DNA.

## Materials and Methods

### Unmodified and B[*a*]P modified DNA constructs

Custom DNA oligonucleotides were purchased from Eurofins MWG Operon and purified by high-performance liquid chromatography (HPLC) using a reverse phase C18 column (all sequences in Supplementary Table S1). Template oligos were Cy3-labeled at an amino-modified C6-dT with a Cy3 NHS ester (GE healthcare) as described^54^. DNA purity was assessed by MALDI-TOF MS.

B[*a*]P-modified template was prepared as described^55^ (Supplementary reaction scheme S1). Briefly, a racemic mixture of (±)-*anti*-B[*a*]PDE (National Cancer Institute Chemical Reference Standard Repository, Kansas City, MO) was incubated with an 11-mer containing a single dG. The reaction yields four isomeric products that were separated by reverse phase HPLC. The selected B[*a*]P-modified 11-mer was ligated to a Cy3-labeled 15-mer using T4 DNA ligase and the final product (26-mer) was purified by reverse phase HPLC with a heated column. The 26-mer unmodified template and B[*a*]P modified template were heat annealed to the appropriate primer depending on the experiment.

### Dpo4 purification and labeling

The *E. coli* strain RW382 was transformed with a plasmid encoding the Dpo4 gene (kindly provided by Roger Woodgate, NICHD). Dpo4 purification was carried as described^44,48^. The native single cysteine in Dpo4 was labeled with a Cy5 maleimide ester (GE healthcare) as described^44^.

### Primer extension and single nucleotide incorporation assays

Cy5-labeled primers and B[*a*]P modified or unmodified templates (15 nM) were incubated with Dpo4 (2 nM for unmodified template and 50 nM for B[*a*]P modified template) in reaction buffer (50 mM Tris-HCl, pH 7.5, 10 mM MgCl_2_, 1 mM DTT, 25 μg/ml bovine serum albumin (BSA), 100 μM dNTPs, (±)10% DMSO) at 37 °C. 10 μL aliquots of the reaction mixture were taken for each time point and mixed with 2x DNA loading buffer (95% formamide, 1 mg/ml bromophenol blue, 10 mM EDTA) to quench the extension reaction. The samples were loaded to a 20% polyacrylamide denaturing gel and run for ~16 hrs at 800 V. Gels were scanned for Cy5 fluorescence in a Typhoon 9210 Variable Mode Imager (GE Healthcare). The gel pictures were generated using ImageJ (https://imagej.nih.gov/ij/) software and the contrast was increased ~10–20% to better visualize all the bands in the gel.

Single nucleotide incorporation assays were carried out at 37 °C in a buffer containing 50 mM Tris-HCl, pH 7.5, 50 mM NaCl, 5 mM Mg, 0.025 mg/ml BSA, 10 mM DTT, 10 nM primer/template. Dpo4 concentration in the both unmodified and B[*a*]P modified template containing reactions was 10 nM. However, incubation time for unmodified template containing reaction was 1 min whereas modified DNA reaction was 20 min. At the end of the incubation time, the reaction was quenched with 2X DNA loading dye. The gel electrophoresis is done similar the primer extension assay.

### Single-molecule experiments and data analysis

DNA was immobilized on a PEGylated quartz slide surface *via* biotin-streptravidin linkage. The PEGylated slide was prepared as previously described^56,57^. Single molecule experiments were done as follows: PEGylated slides were incubated with a streptavidin solution (0.02 mg/ml) for 7 min. After washing, 10–15 pM biotinylated primer-template was introduced and incubated for another 7 min. After a final wash, Cy5-labeled Dpo4 (10–15 nM) was introduced in binding buffer containing 50 mM Tris-HCl, pH 7.5, 3.5 mM CaCl_2_, 25 μg/ml BSA, oxygen scavenging system (dihydroxybenzoic acid and photocatechuate dioxygenase from *Pseudomonas sp*.) and 1 mM Trolox. Calcium ions were used for all smFRET experiments to prevent DNA synthesis in the presence of dNTPs. Single molecule experiments were conducted at 21 °C in a home-built prism-based total internal reflection microscope. All the smFRET experiments were carried out at 80 ms time resolution. The traces were analyzed with a 5-point moving average; a few un-averaged trajectories are shown in Supplementary Fig. S7. The apparent FRET efficiencies were calculated as FRET = I_A_/(I_D_ + I_A_), where I_D_ and I_A_ are the of donor and acceptor fluorescence intensities, respectively. The anti-correlated changes in the donor and acceptor channels are not necessarily of the same magnitude. This is not uncommon when the environment of the dyes changes in the different conformations. However, this is not an issue in this work because we do not determine accurate distances from our FRET measurements, we simply use the observed FRET values as identifiers of the different conformations. Signal fluctuations that show no anti-correlation are not included in our analysis. All smFRET histograms were built using >100 trajectories, and fit to single or double Gaussian curves (Supplementary Fig. S8). The calculated FRET values are associated with an error of ±0.02. The transition density plots were created using HHM fit^58^. Kinetic rate constants were determined by fitting dwell time histograms to single exponential decays (Supplementary Fig. S2), as described^59^. Relative changes in stabilization energies were calculated as ∆∆G = −RT ln(k_off,2_/k_off,1_), as described^60^.

### MD simulations

The crystal structure of the binary complex of Dpo4 (pdbid 2IA6^25^) with the B[*a*]P adduct was used as the starting template for all simulations. MolProbity^61^ was employed to check and protonate the structure. The DNA substrate sequence was modified to be similar to the primer used experimentally (5′-CATGGGTATC, 3′-GTACCCATA(B[*a*]P-*cis*-G)ATA), and the adduct in the crystal structure was modified from the trans conformation to the cis conformation, with the backbone of the DNA helix remaining in the same location as the crystal structure. The final C base in the 5′ DNA strand was also modified to investigate several different systems and structural aspects. A total of nine systems with different nucleotide substitutions, adduct orientations and solvents were created to investigate the impact of DMSO on the system—six were in pure water, three were in 90% water and 10% DMSO. All molecular dynamics simulations and structural analysis was done with the AMBER 14 simulation package^62–64^, and PCA and NMA were done with in the ProDy module in VMD^65,66^. Further specific details of the computational simulations can be found in the Supporting Information.

## Electronic supplementary material

Supplementary Materials
