# Supplementary material for: Bulky Lesion Bypass Requires Dpo4 Binding in Distinct Conformations
Source: Sci Rep. 2017 Dec 12;7:17383. doi: 10.1038/s41598-017-17643-0 (PMC5727293; doi:10.1038/s41598-017-17643-0)
Supplement: Supplementary file 1 — Supplementary Materials [file 41598_2017_17643_MOESM1_ESM.pdf]

Supporting information for

Bulky Lesion Bypass Requires Dpo4 Binding in Distinct Conformations

Pramodha S. Liyanage[1], Alice R. Walker[2], Alfonso Brenlla[1], G. Andrés Cisneros[2], Louis J. Romano[1]* and David Rueda[3,4]*

[1] Department of Chemistry, Wayne State University, Detroit, MI 48202, USA.

[2] Department of Chemistry, University of North Texas, Denton, TX, 76201, USA.

[3] Molecular Virology, Department of Medicine, Imperial College London, Du Cane Road, London W12 0NN, UK

[4] Single Molecule Imaging Group, MRC London Institute of Medical Sciences (LMS), Du Cane Road, London W12 0NN, UK

Contents

1. Supplementary methods S2
2. Supplementary figures S3
3. Supplementary table S13
4. Reaction scheme S14
5. References S15

Supplementary Methods

Principal Component Analysis. Principal component analysis, or PCA, can be a useful tool to isolate and show large protein movements. PCA was performed on 33,000 snapshots for each trajectory. 100 total modes were generated, and the cross-correlation plots for those modes were subtracted in the same way described in above for the distance correlation plots (Fig. S9). These plots show some interesting features and strong differences; notably, there are again fewer differences between B[*a*]P-dG:dC in water and B[*a*]P-dG:dG in water versus B[*a*]P-dG:dC in water and B[*a*]P-dG:dC in DMSO. In light of the experimental results that show a return to function for Dpo4 with the adduct in a solvent-exposed conformation, the substantial differences in the large motions of the protein support the concept that not only is the structure of the protein different, but that the overall movement of the protein has drastically changed. A representative depiction of the first (and largest) principal coordinate (PC) for B[*a*]P-dG:dC in water and B[*a*]P-dG:dG in DMSO can be seen in Fig. S8.a and Fig. S8.b respectively. The two modes contain not only very different movements, but also the B[*a*]P-dG:dC in water's first PC has substantially less movement overall. This is also consistent with the experimental result that when the adduct is stacked within the major groove of the DNA helix, the protein is blocked from functioning properly. That said, it is important to recognize that the first PC only represents one large-scale motion of the protein—while more difficult to visually interpret, the overall correlation differences are more telling, since they show substantial differences in many of the PCs rather than just one.

MD simulations. The simulations in pure water comprise two binary insertion complexes and one binary pre-insertion complex. All of the insertion binary complexes in water have the 3'-B[*a*]P adduct stacked within the DNA helix and the modified G nucleobase stacked within the minor groove[1]. The two sub-systems for the insertion binary complex had modified bases at the ending 5' position opposite the adduct, which was changed from C to G in the second system. The corresponding nucleobase was deleted completely from the third to simulate the pre-insertion complex. These systems were compared in order to investigate the effect of different nucleobases on the stability of the complex. Two additional simulations were run in 10% DMSO with the adduct flipped out into solvent; one with the original experimental primer given above and one with the final nucleobase at the end of the 5' strand (across from the adducted base location) deleted. Further changes were not performed for the DMSO since the flipped-out conformation for the B[*a*]P-cis-G adducted base does not have substantial interaction with the corresponding nucleobase. Additionally, the TYR-274 amino acid in the protein chain was changed to the primary Dunbrack rotamer (53.1377%) in order to eliminate steric clashes with the flipped out DNA base in the 10% DMSO systems[2].

Partial charges for the B[a]P-cis-G adduct were calculated using RESP fitting on the RED development servers[3,4] and the standard procedure for creating modified nucleotides for proper linkages between nucleobases. An additional angle parameter was

taken from Mocquet 2007, and the partial charges generated in this work compared favorably with the partial charges from those results (all calculated charges and other parameters are reported in the supporting information)[5]. The guanine geometry parameters were taken from the OL15 DNA force field[6], and the remaining geometry parameters for the adduct were generated from the GAFF force field[7]. Parameters for the Ca^{2+} ions present in the crystal structure[8] and parameters for the DMSO solvent box[9] (where appropriate) were taken from the literature.

Molecular dynamics simulations on all systems were performed with the pmemd.cuda program from AMBER16, with the ff14SB force field for all of the protein parameters,[10-12] the OL15 force field for the DNA parameters[6] and a 1 fs time step. Long-range electrostatics were treated with sPME with an 8Å cutoff for all nonbonded interactions.[13] All simulations were performed in the NVT ensemble with the Berendsen thermostat and barostat[14], and SHAKE was applied to all bonds involving hydrogen atoms.

Prior to solvation, all systems were neutralized to a net charge of 0 with Na^+ ions in AMBER16's tleap program. The water systems were solvated in a rectangular box of TIP3P water using a 12Å pad between the surface of the protein and the edge of the box[15]. For the systems with 10% DMSO, in order to ensure an even distribution of DMSO molecules within the box, the AMBER16 program AddToBox was used to generate the 10% DMSO/90% TIP3P water solvent boxes, with the parameters of the box given to meet or exceed 12Å from the surface of the protein and the edge of the box. RMSD, RMSF, correlation analysis, and solvation shell tracking were all calculated with AMBER16's cpptraj program. PCA and NMA were done with in the ProDy module in VMD[16,17]. 33,000 snapshots from each trajectory were analyzed with ProDy's PCA algorithm, with 100 PC modes generated.

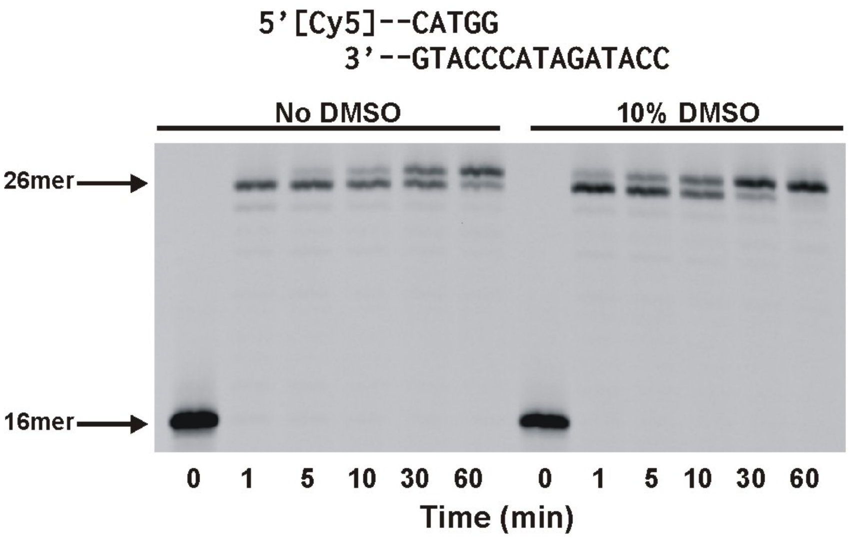

Figure S1. Dpo4 running start primer extension assay with unmodified template. The 16mer primer is fully extended to 26mer product in one minute. The Dpo4 concentration is 50 nM. The sequence used for the extension assay is shown on the top of the gel and in Table S1.

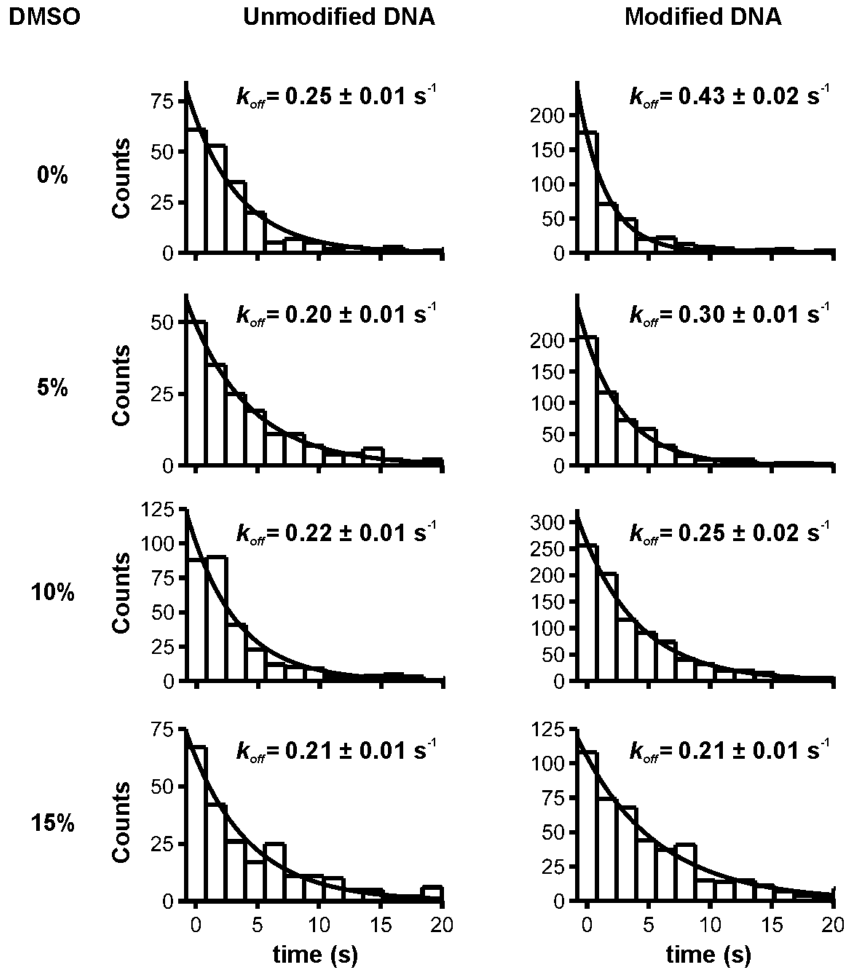

Figure S2. Dwell time distributions for Dpo4-DNA binary complex. The dissociation constants (k_{off}) were calculated by fitting data to single exponential decays. The corresponding smFRET experiments were carried out as a function of DMSO concentration shown in Fig. 3c.

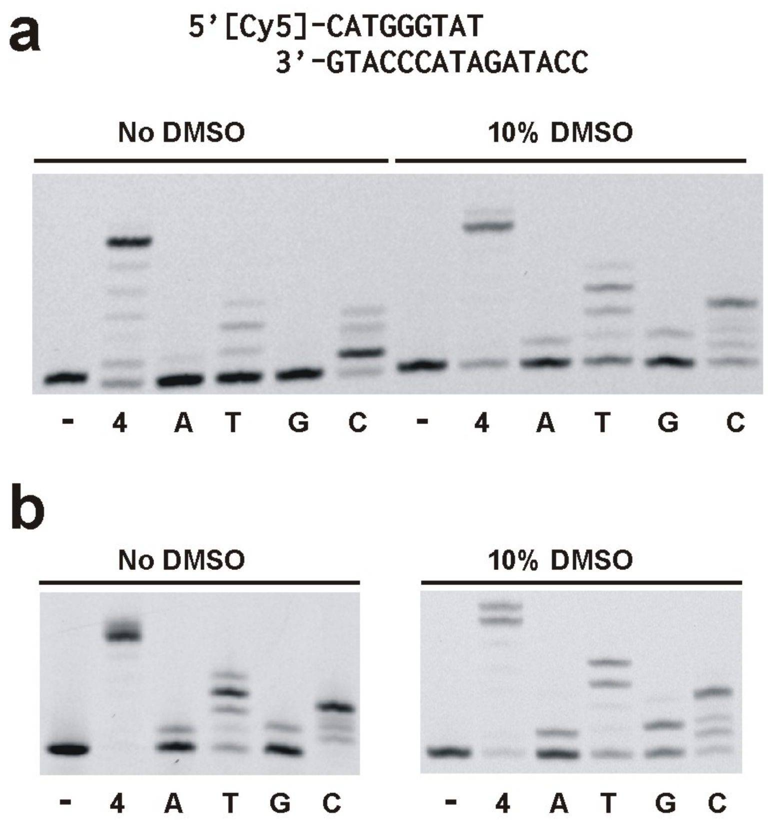

Figure S3. Single nucleotide incorporation assay for unmodified template. **(a)** Dpo4 primarily incorporates the next correct nucleotide in the unmodified DNA construct. The gel picture shows the tendency of dNTP incorporation across the dG, templating base in the 20mer/26mer primer-template. Unmodified DNA sequence used for this assay is shown on the top. Dpo4 mainly incorporates dC in both in absence and presence of DMSO. Lanes 1 and 7 represent control experiments in absence of dNTPs in the experiments (labeled as -). Lanes 2 and 8 represent experiments with all 4 dNTPs (labeled as 4). Other lanes contain only the designated dNTP. The Dpo4 concentration is 10 nM and the incubation time is 1 min. **(b)** This is the same reaction as described in (**a**) but the incubation time is 10 min.

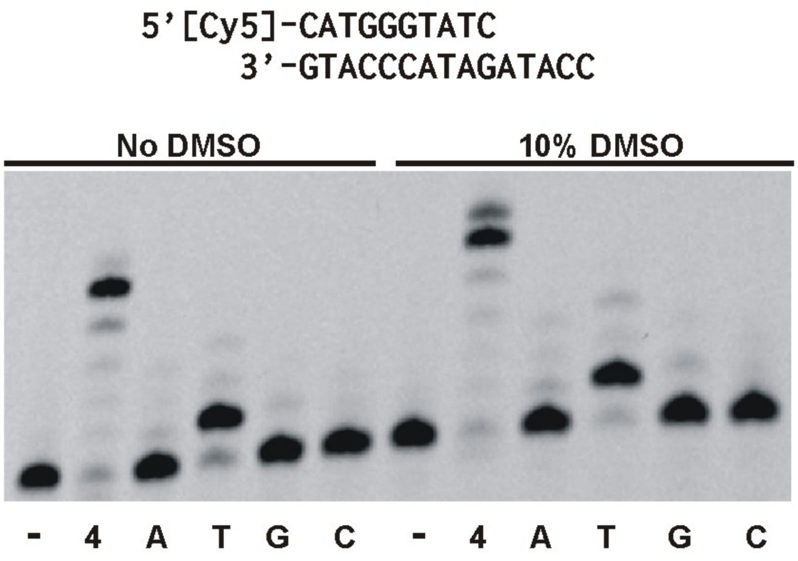

Figure S4. Single nucleotide incorporation assays on 21mer/26mer unmodified DNA primer-template (Table S1). Cy5-labeled DNA primer shows the extended products on the gel. Dpo4 incorporates the next correct dNTP, dT in the DNA construct shown on top of the gel. In this single nucleotide incorporation assay, lanes 1and 7 (dash line) corresponding to control experiments without dNTPs. Lanes 2 and 8 contain all four dNTPs (labeled as 4). Other lanes contain the designated dNTP below the lane. The primer extension reaction was quenched after 1 min.

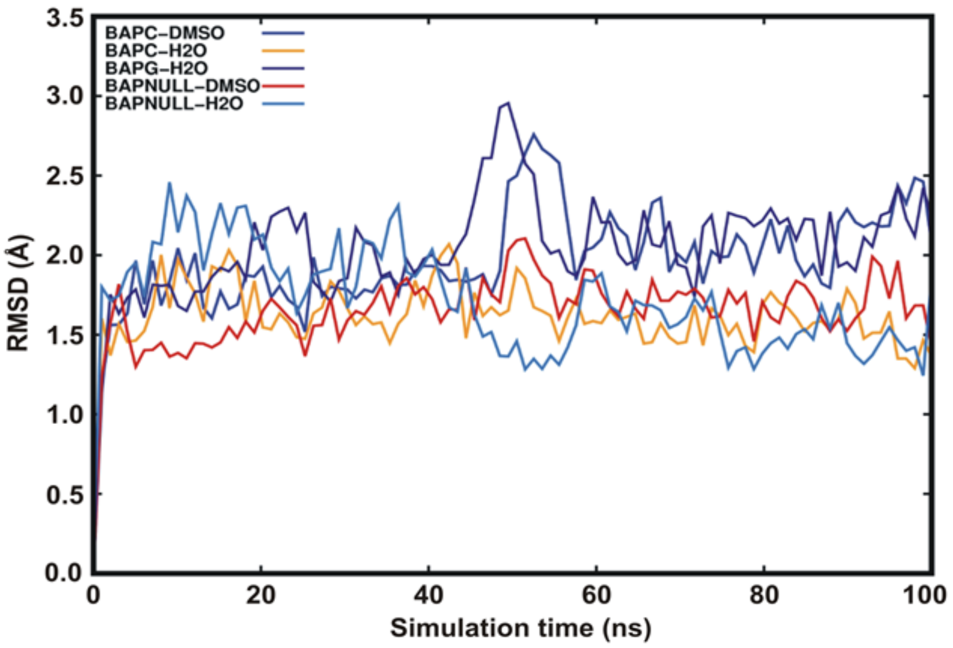

Figure S5. The RMSD of the protein backbone over time for each trajectory.

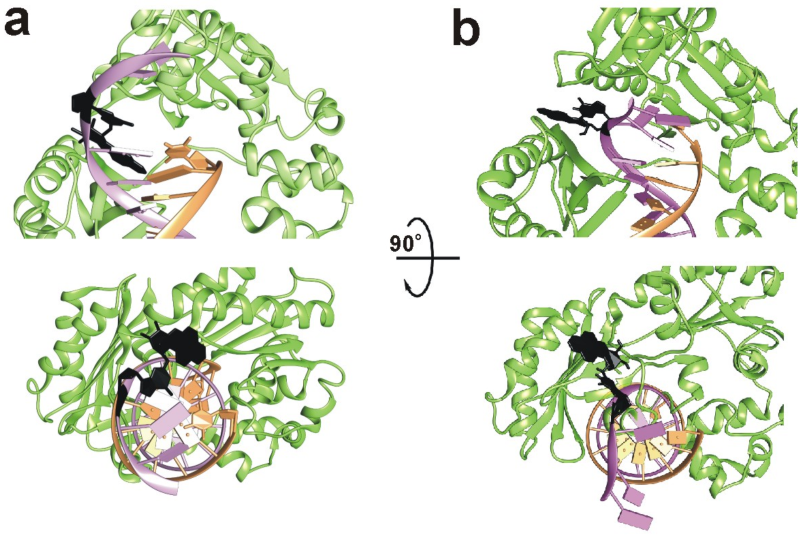

Figure S6. Comparison of the adduct conformation in the presence and absence of DMSO. **(a)** A representative structure of Dpo4-DNA binary complex. The adduct is stacked in the minor groove. **(b)** A representative structure of Dpo4-DNA binary complex in the presence of DMSO. The adduct is flipped outside of the helix and exposed to the solvent.

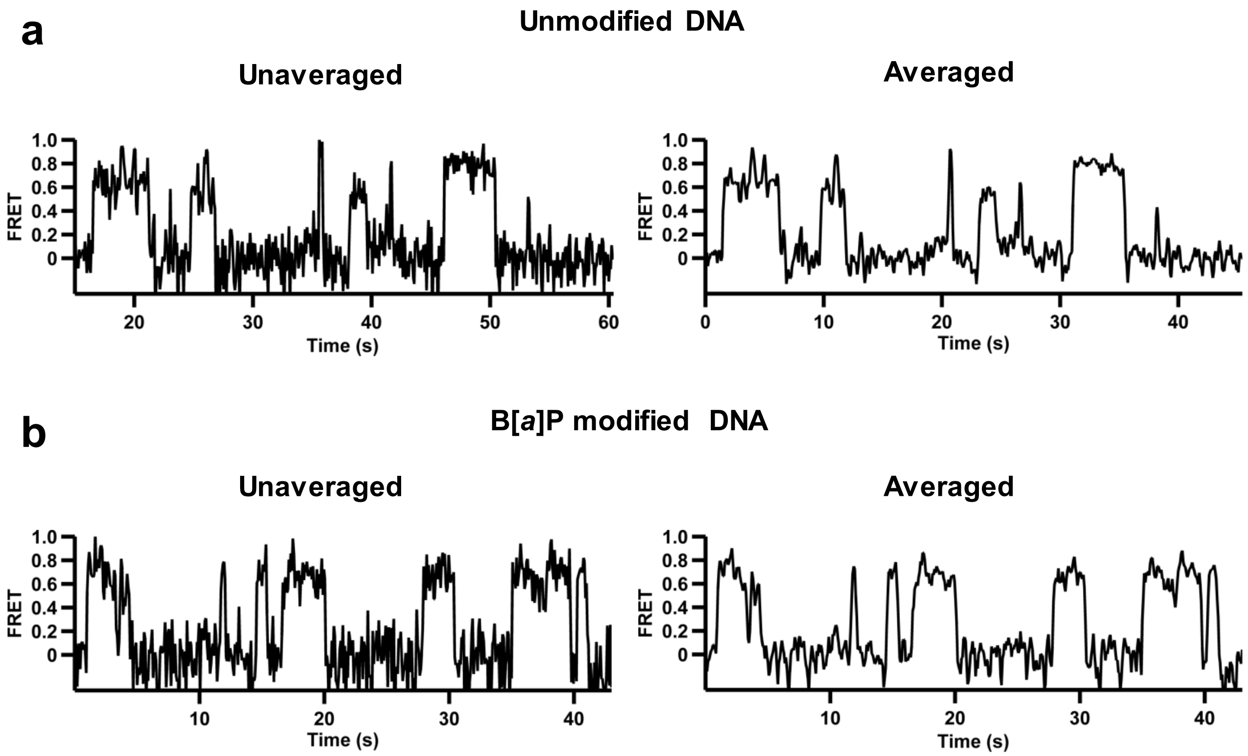

Figure S7. Characteristic FRET traces for the DNA-Dpo4 binary complex. **(a)** Sample trajectories for the unmodified template (Fig. 2b) and **(b)** B[*a*]P modified template, binary complexes (Fig. 2c). Unaveraged and 5-point averaged traces are shown in the left and right columns respectively.

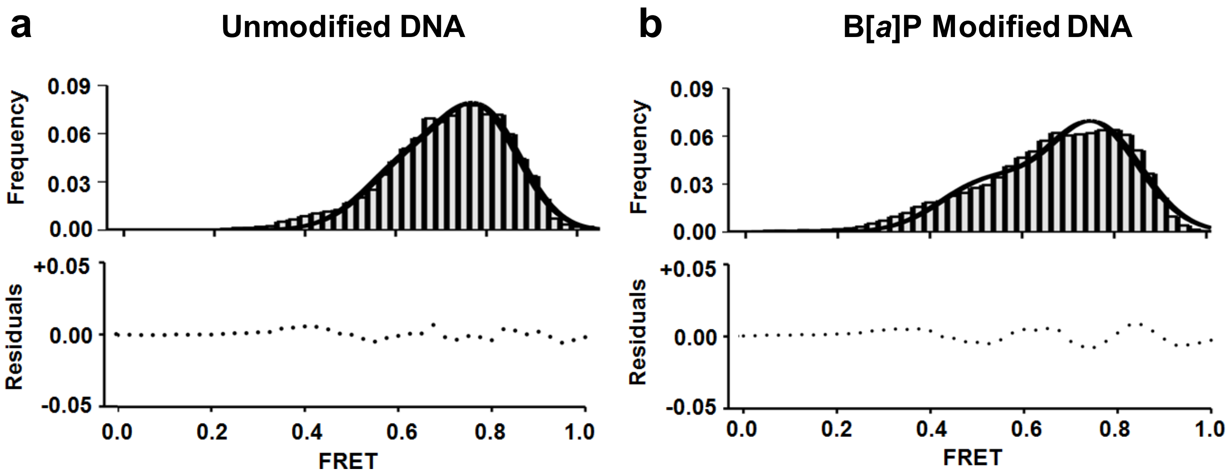

Figure S8. Fitting residuals for the FRET distributions in Fig. 2b (a) and 2c (b).

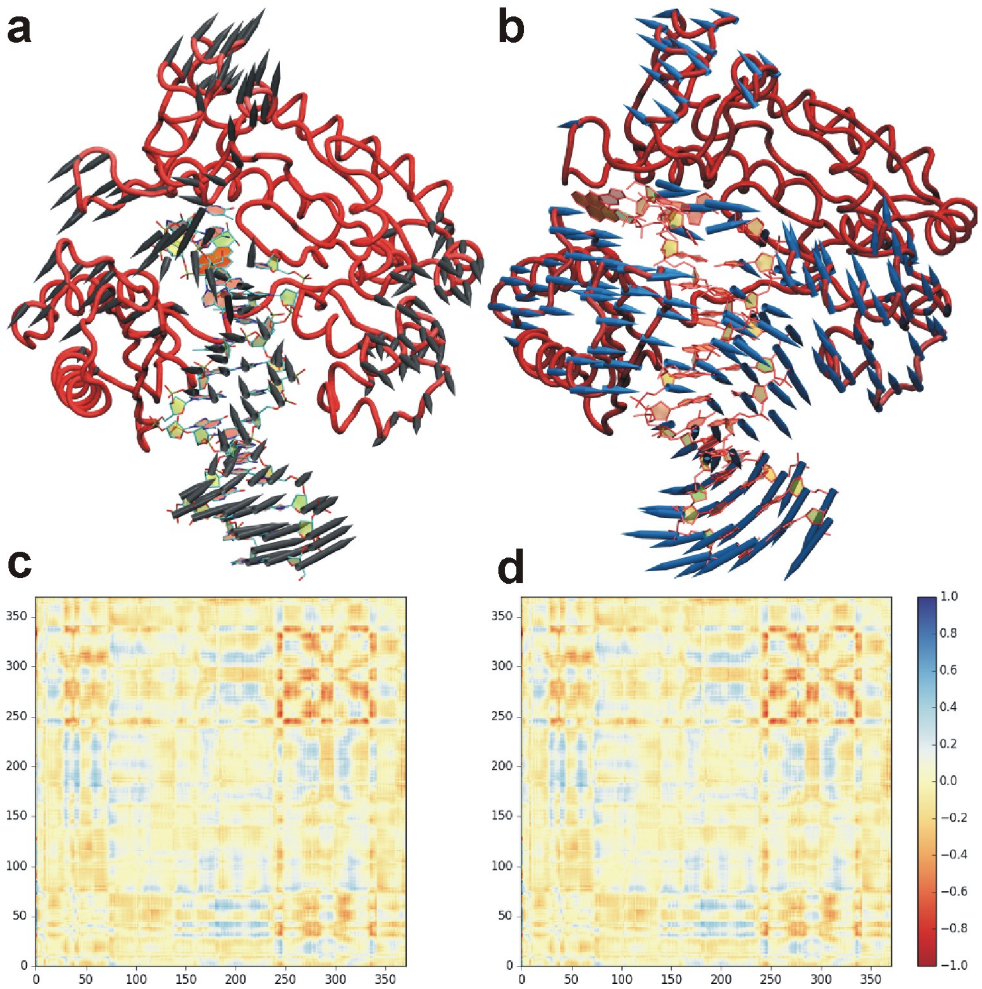

Figure S9: Structural representations from principal coordinate analysis and difference correlation matrices generated from the first 100 PC modes and normalized to +/- 1. **(a) T**he first principal coordinate (PC) mode of B[*a*]P-dG:dC in water**. (b)** The first PC mode of B[*a*]P-dG:dG in DMSO in the solvent-exposed orientation. **(c)** The subtraction of the normal mode correlation matrices of B[*a*]P-dG:dC in water from B[*a*]P-dG:dG in water **(d)** The subtraction of the normal mode correlation matrices of B[*a*]P-dG:dC in DMSO subtracted from B[*a*]P-dG:dC in water.

Table S1. List of DNA sequences used in this study.

Experiment	Name	Sequence[1]
Synthesis of B[*a*]P template	11mer	5'-CCA TAG ATA CC-3'
	15mer	5'-CpAT GTC GTT TTG GTG-3 '
	26mer scaffold	5'-CAC CAA AAC GAC ATG GGT ATC TAT GG-3'
smFRET	20mer primer	5' [Biotin]-CAC CAA AAC GAC ATG GGT AT-3'
	21mer primer	5' [Biotin]-CAC CAA AAC GAC ATG GGT ATC-3'
	26mer modified template	5'-CCA TAG ATA CCC ATG TCG TTT TGG TG-3'
	26mer unmodified template	5'-CCA TAG ATA CCC ATG TCG TTT TGG TG-3'
Primer extension	16mer primer	5' [Cy5]-CAC CAA AAC GAC ATG G-3'
	20mer primer	5' [Cy5]-CAC CAA AAC GAC ATG GGT AT-3'
	21mer primer	5' [Cy5]-CAC CAA AAC GAC ATG GGT ATC-3'

[1] Benzo[a]pyrene is linked to the red **G**, and Cy3 to an amino-linker on the underlined **T**.

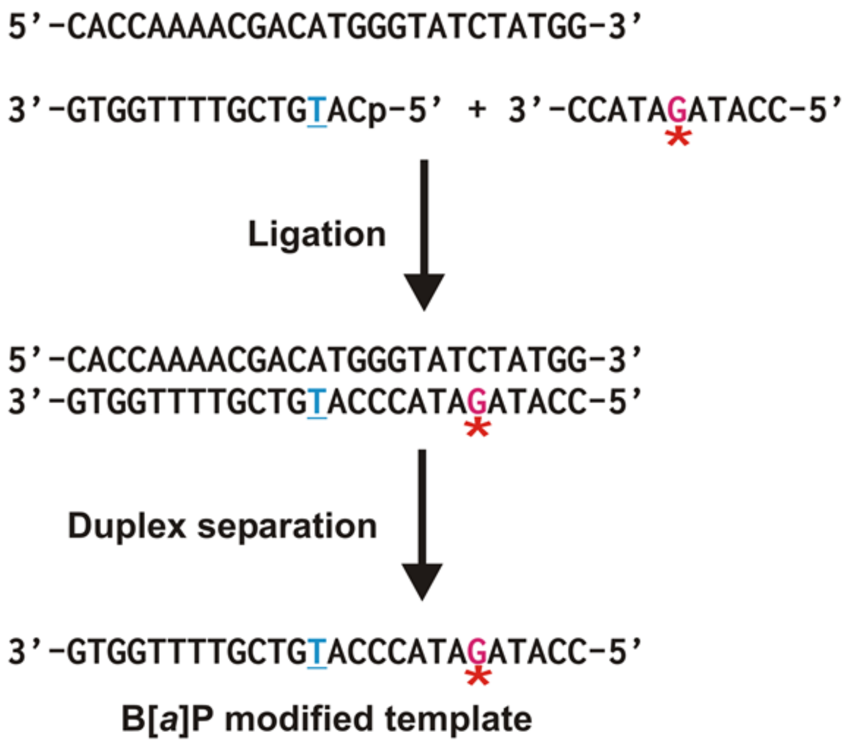

Reaction scheme S1. Synthesis of (+)-*cis*-B[*a*]P-N^2-dG modified 26mer template.

References

1 Mu, H. *et al.* Role of structural and energetic factors in regulating repair of a bulky DNA lesion with different opposite partner bases. *Biochemistry* **52**, 5517-5521 (2013).

2 Shapovalov, M. V. & Dunbrack, R. L., Jr. A smoothed backbone-dependent rotamer library for proteins derived from adaptive kernel density estimates and regressions. *Structure* **19**, 844-858 (2011).

3 Dupradeau, F. Y. *et al.* The R.E.D. tools: advances in RESP and ESP charge derivation and force field library building. *Phys Chem Chem Phys* **12**, 7821-7839 (2010).

4 Vanquelef, E. *et al.* R.E.D. Server: a web service for deriving RESP and ESP charges and building force field libraries for new molecules and molecular fragments. *Nucleic Acids Res* **39**, W511-517 (2011).

5 Mocquet, V. *et al.* The human DNA repair factor XPC-HR23B distinguishes stereoisomeric benzo[a]pyrenyl-DNA lesions. *Embo j* **26**, 2923-2932 (2007).

6 Cheatham, T. E., 3rd & Case, D. A. Twenty-five years of nucleic acid simulations. *Biopolymers* **99**, 969-977 (2013).

7 Wang, J., Wolf, R. M., Caldwell, J. W., Kollman, P. A. & Case, D. A. Development and testing of a general amber force field. *Journal of Computational Chemistry* **25**, 1157-1174 (2004).

8 M. Bradbrook, G. *et al.* X-Ray and molecular dynamics studies of concanavalin-A glucoside and mannoside complexes Relating structure to thermodynamics of binding. *Journal of the Chemical Society, Faraday Transactions* **94**, 1603-1611 (1998).

9 Fox, T. & Kollman, P. A. Application of the RESP Methodology in the Parametrization of Organic Solvents. *The Journal of Physical Chemistry B* **102**, 8070-8079 (1998).

10 AMBER 2016 (University of California, San Francisco, 2016).

11 Salomon-Ferrer, R., Götz, A. W., Poole, D., Le Grand, S. & Walker, R. C. Routine Microsecond Molecular Dynamics Simulations with AMBER on GPUs. 2. Explicit Solvent Particle Mesh Ewald. *Journal of Chemical Theory and Computation* **9**, 3878-3888 (2013).

12 Maier, J. A. *et al.* ff14SB: Improving the Accuracy of Protein Side Chain and Backbone Parameters from ff99SB. *Journal of Chemical Theory and Computation* **11**, 3696-3713 (2015).

13 Essmann, U. *et al.* A smooth particle mesh Ewald method. *The Journal of Chemical Physics* **103**, 8577-8593 (1995).

14 Berendsen, H. J. C., Postma, J. P. M., van Gunsteren, W. F., DiNola, A. & Haak, J. R. Molecular dynamics with coupling to an external bath. *The Journal of Chemical Physics* **81**, 3684 (1984).

15 Jorgensen, W. L., Chandrasekhar, J., Madura, J. D., Impey, R. W. & Klein, M. L. Comparison of simple potential functions for simulating liquid water. *The Journal of Chemical Physics* **79**, 926-935 (1983).

16 Bakan, A., Meireles, L. M. & Bahar, I. ProDy: Protein Dynamics Inferred from Theory and Experiments. *Bioinformatics* **27**, 1575-1577 (2011).

17 Humphrey, W., Dalke, A. & Schulten, K. VMD: Visual molecular dynamics. *Journal of Molecular Graphics* **14**, 33-38 (1996).
